# Effects of an aged tissue niche on the immune potency of dendritic cells using simulated microgravity

**DOI:** 10.1038/s41514-023-00111-7

**Published:** 2023-07-01

**Authors:** Mei ElGindi, Jiranuwat Sapudom, Anna Garcia Sabate, Brian Chesney Quartey, Aseel Alatoom, Mohamed Al-Sayegh, Rui Li, Weiqiang Chen, Jeremy Teo

**Affiliations:** 1 Laboratory for Immuno Bioengineering Research and Applications, Division of Engineering, New York University Abu Dhabi, Abu Dhabi, PO Box 129188, United Arab Emirates; 2 Biology Division, New York University Abu Dhabi, P.O. Box 129188, Abu Dhabi, United Arab Emirates; 3 Department of Biomedical Engineering, New York University, 6 MetroTech Center, Brooklyn, NY 11201 USA; 4 Department of Mechanical and Aerospace Engineering, New York University, 6 MetroTech Center, Brooklyn, NY 11201 USA

**Keywords:** Ageing, Cell biology

## Abstract

Microgravity accelerates the aging of various physiological systems, and it is well acknowledged that aged individuals and astronauts both have increased susceptibility to infections and poor response to vaccination. Immunologically, dendritic cells (DCs) are the key players in linking innate and adaptive immune responses. Their distinct and optimized differentiation and maturation phases play a critical role in presenting antigens and mounting effective lymphocyte responses for long-term immunity. Despite their importance, no studies to date have effectively investigated the effects of microgravity on DCs in their native microenvironment, which is primarily located within tissues. Here, we address a significantly outstanding research gap by examining the effects of simulated microgravity via a random positioning machine on both immature and mature DCs cultured in biomimetic collagen hydrogels, a surrogate for tissue matrices. Furthermore, we explored the effects of loose and dense tissues via differences in collagen concentration. Under these various environmental conditions, the DC phenotype was characterized using surface markers, cytokines, function, and transcriptomic profiles. Our data indicate that aged or loose tissue and exposure to RPM-induced simulated microgravity both independently alter the immunogenicity of immature and mature DCs. Interestingly, cells cultured in denser matrices experience fewer effects of simulated microgravity at the transcriptome level. Our findings are a step forward to better facilitate healthier future space travel and enhance our understanding of the aging immune system on Earth.

## Introduction

Aged individuals and astronauts participating in manned space flights have several commonalities, a major one being a compromised immune system. A hallmark sign of an aged immune system is increased susceptibility to viral and bacterial infections^1^. Similarly, since the beginning of manned space flight, it has been documented that astronauts returning from missions due to several physiological stressors, in particular microgravity (µG), also suffer from compromised immune systems^2^. Therefore, studies conducted to determine the effects of µG on the immune system will not only facilitate future and longer duration space missions through de novo countermeasures, but it has also been widely acknowledged that such studies will also help us understand our immune system as we age here on Earth^3,4^. Early studies on the effects of µG on immune cells were limited to in vitro analysis of blood samples obtained from astronauts before and after flights, where they found decreased inflammatory cytokines and reduced T lymphocyte (T cell) counts^5^. For decades, researchers have pursued to elucidate the cause of compromised immunity in astronauts, with meager advancement in knowledge, perhaps due to experimental and technical limitations that plague space biology research. In the last half-decade, vast resources have been made available by various funding agencies (NSF, NIH), national space agencies (NASA, JAXA, ESA), and the International Space Station National Laboratory (ISS) to use their orbiting platforms to accelerate aging not only on immune cells but also on a plethora of cell and organoid systems for research studies to benefit health on Earth.

In vitro, biological research performed onboard orbital space missions would be the most relevant. However, the results will be a combinatorial effect of the myriad of environmental stressors associated with space missions and not possible to decouple. As space biology was, and still is, complicated and costly, researchers developed platforms on Earth that reproduce these space environment stressors separately. µG reproduced by various platforms on Earth can be real or simulated (sµG), with each platform having differing capabilities in terms of µG magnitudes and duration of exposure that ranges from seconds to months^2^. It has been deduced that random positioning machines (RPMs) are the most appropriate for long-term space biology studies and, therefore, for aging studies as well^2,6,7^.

The attenuation of the immune response found in the elderly or astronauts could be, in part, due to the dysregulation of dendritic cells (DCs). DCs play a crucial role in bridging the innate and adaptive immune systems. DCs are the most potent antigen-presenting cells (APCs) of the innate immune system and present processed foreign peptides on major histocompatibility complexes (MHCs) that allow them to trigger cells of the adaptive immune system. Therefore, dysregulation of DCs or their immune functions could manifest in the previously reported inadequate immune potency^8–10^. Considering their importance in mounting an effective and long-lasting immune response, little work has been done to investigate how µG affects DC differentiation, maturation, and associated function^2^. Previously, sµG was shown to decrease the differentiation abilities of DCs and reduce their immunogenicity in terms of T-cell activation^8^. Another study showed that under sµG conditions, DCs had lower expression of maturation surface markers, HLADR (also known as MHCII), and CD80, as well as a diminished ability to uptake pathogens^11^. Studies are urgently needed to confirm the aforementioned findings and fully understand the molecular and cellular aspects involved during the exposure of DCs to microgravity.

While in vitro space biology experiments have provided us with knowledge, albeit limited, these are primarily studies conducted on cells in 2D environments. The caveat is that petri dishes inaccurately mimic in vivo states. Recently, 3D cell culture models have become increasingly utilized to better mimic a cell’s native microenvironment, and immune cells, in particular, have been found to be sensitive to changes in the biophysical cues found in their microenvironment^12–15^. As DCs are primarily resident in tissue and not in circulation, using biomimetic 3D cell culture models will provide better insights into the effects of µG on these cells.

To perform comprehensive cell biology on RPMs, with ample samples for statistical confidence and simultaneously having appropriate static controls and sµG exposure using the same batch of cells, engineered microvessels exist^16^. Our published microvessel design reduces the need for copious amounts of reagents; ensures that samples are kept at the axis of the rotating frames of the RPM, thereby maintaining the quality of sµG; and eliminates the need for sealing to prevent culture media leaks, thus facilitating the gaseous transfer. These microvessels have been used successfully for the evaluation of fibroblasts and T-cell biology, cultured within 3D collagen hydrogels and exposed to RPM sµG^17,18^.

We now have the opportunity to comprehensively investigate DC immune biology using physiologically relevant 3D cell culture models, having addressed the limitations of RPM-induced sµG via engineered solutions. It has been reported that aging tissues have higher porosity, decreased collagen fibril density, and extensive breakdown in crosslinking that is attributed to loss of normal tissue homeostasis where degradation supersedes synthesis^19–21^. Hence, in this study, we also investigated the effects of sµG on THP-1-derived DCs embedded in fibrillar collagen I matrices of different densities to mimic loose and dense tissue microenvironments.

Our work provides a multiresolution assessment of applying sµG to simulate accelerated aging on DCs in a 3D biomimetic cellular niche recapitulating normal and aged tissue. We analyzed surface marker expression and cytokine secretion for maturity and proinflammatory signatures, functional assays to fully characterize DCs exposed to RPM conditioning, and high-throughput RNA sequencing for genome-wide profile analyses. We assessed iDC function through uptake or phagocytic activity and that of mDCs by the ability to activate T-cells in coculture conditions as an indication of innate-adaptive communication.

## Results

DCs are key mediators between the innate and adaptive immune systems. They differentiate from monocytes and can be classified by their maturation stages. Immature DCs (iDCs) in peripheral tissues such as the skin, lungs, or lymphoid tissues seek out foreign antigens, are characterized by low levels of MHC and costimulatory molecules (e.g., CD80 and CD86) on their surface, and have poor ability to stimulate a T-cell response^22^. iDCs that uptake and process foreign antigens are said to be activated and differentiate into mature DCs (mDCs). mDCs have increased expression of MHC and costimulatory molecules on their surface and secrete specific cytokines, such as IFNγ and TNFα, that allow them to effectively stimulate the differentiation and proliferation of T-cells of the adaptive immune system at the lymph nodes^22^. Primarily, DCs are found in tissues such as the dermis or in lymph nodes, and yet, all studies conducted on the effects of RPM exposure on DCs utilize traditional in vitro 2D cell culture with cell lines^8,9,23^.

**Fig. 1:**
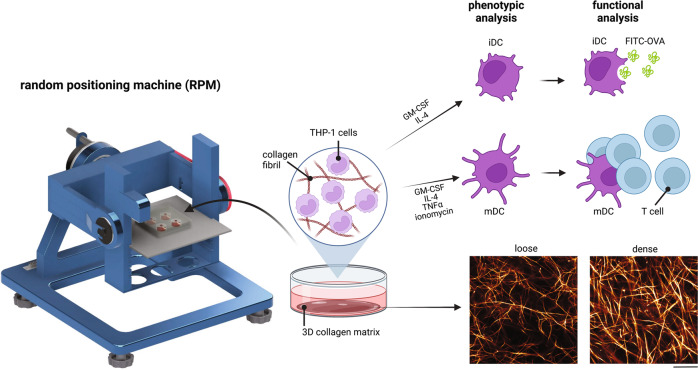
Experimental design to study DC differentiation from monocytes under the effects of matrix density and RPM conditioning. Monocytes (THP-1) were cultured with iDC or mDC differentiation cocktail in 1 mg/mL (loose) and 3 mg/mL (dense) 3D collagen matrices either under static or RPM conditions for 3 days. iDCs and mDCs were then assessed for phenotypic changes by analysis of surface markers, cytokine secretion, and RNA sequencing. Following this differentiation, FITC-OVA was added to iDCs for 1 h under the same conditions to assess functional uptake ability. T cells were cocultured with mDCs for an additional 3 days, either under static or RPM conditions to determine the effects on mDC function in terms of activating T cells and changes in cytokine secretion. The scale bar represents 25 µm.

In this work, we investigated the effects of accelerated aging, via exposure to RPM conditioning, on the differentiation of THP-1-derived monocytes into iDCs and subsequent maturation into mDCs in 3D fibrillar collagen matrices that better mimic the native cellular microenvironment. We utilized loose (1 mg/mL; pore size of 11.07 ± 0.62 µm) and dense (3 mg/mL; pore size of 3.74 ± 0.75 µm) collagen matrices to further determine how aged and normal tissue density plays a role in DC differentiation and maturation. The pore size range used in this study is well correlated to those presented in native interstitial tissues^14^. Specifically, we determined changes in DC-specific surface markers, the cytokine secretion profile, hallmark cellular functions, and the transcriptome profile of these cells. A schematic illustration of the experimental design and setup, as well as matrix images, can be found in Fig. 1.

### Immature DCs (iDCs)

To determine the effects of accelerated aging on DC differentiation in loose and dense matrices, THP-1 cells, a monocyte cell line, were differentiated into iDCs for 3 days in the presence of GM-CSF and IL-4 using static culture as a control and onboard the RPM. The cells were then assessed for changes in surface markers, cytokine secretion profile, antigen uptake ability, and global gene expression changes using RNA sequencing.

### Dense matrix supports while RPM conditioning reduced iDC differentiation capabilities

**Fig. 2:**
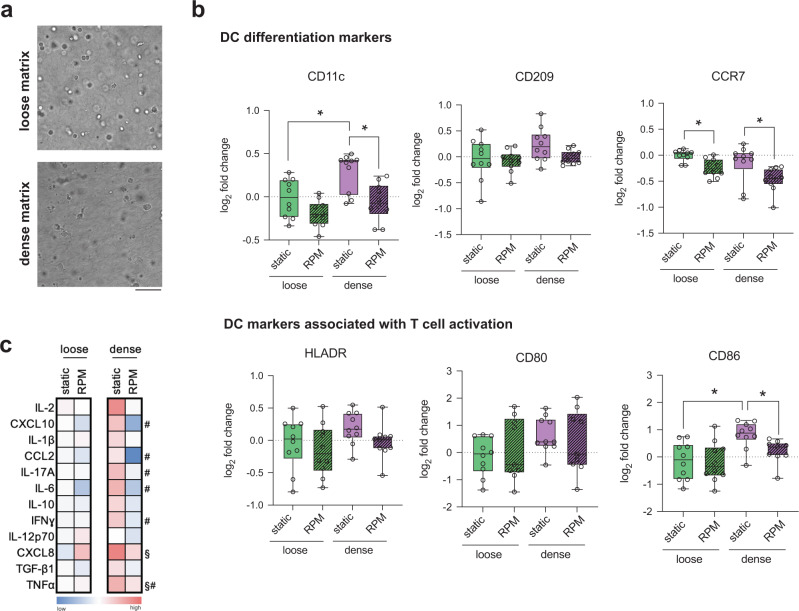
Quantitative analysis of iDC surface markers and cytokines. Monocyte-derived iDCs were cultured for 3 days under static conditions or on the RPM in loose and dense matrices. **a** Representative brightfield images of iDCs cultured in loose and dense matrices under static conditions. The scale bar represents 100 µm. **b** Analysis of surface markers associated with differentiation, namely, CD11c, CD209, and CCR7, and markers associated with T-cell activation, namely, HLADR, CD80, and CD86, using flow cytometry. The log_2_-fold change in geometric mean fluorescence intensity (gMFI) was calculated relative to samples cultured in loose matrices under static conditions. **c** Heatmap of the log_2_-fold change in cytokine secretion by iDCs cultured in loose and dense matrices under static and RPM conditions. The log_2_-fold change in the median concentration of cytokines (pg/mL) was calculated relative to the cytokine concentration secreted by iDCs in loose matrices under static conditions. Experiments were performed with at least four replicates. The box and whiskers graphs used have the center line at the median value. The upper and lower bounds of the box extend from the 25th to 75th percentiles, and the whiskers are plotted to the minimum and maximum values. * indicates significance at *p* ≤ 0.05. For the heatmap, # indicates a significant *p* ≤ 0.05 of samples in dense matrices under RPM conditions compared to dense matrices under static conditions. § indicates significant *p* ≤ 0.05 of samples in dense matrices at static conditions compared to loose matrices at static conditions.

Following a 3-day differentiation into iDCs, cell morphology was first visualized using a bright field microscope. Figure 2a shows that in loose matrices under static conditions, cells appear to be rounder and more symmetrical than those cultured in dense matrices. We observed no morphological differences between samples cultured in static conditions and on the RPM. Next, iDCs were characterized through surface marker expression, namely, CD11c, CD209, CCR7, HLADR, CD80, CD86, and CD206. A representative gating strategy for these markers is found in Supplementary Figure 1. We first analyzed surface markers that are associated with DC differentiation. Under static culture conditions, CD11c, the most common DC cell surface marker, was found to be expressed at significantly higher levels in dense cell culture matrices relative to those cultured in loose matrices (Fig. 2b)^24^. Upon exposure to RPM conditioning, there were no significant differences in CD11c expression of iDCs in loose and dense matrices, but the expression was significantly reduced compared to their static controls for iDCs in denser matrices (Fig. 2b). CD209, a DC cell adhesion, and pathogen recognition receptor did not show any significant changes in levels in all culture conditions (Fig. 2b)^25^. Compared to cells cultured statically, chemokine receptor CCR7, critical for DC homing to lymph nodes, was significantly reduced in both loose and dense matrices upon exposure to RPM conditions, whereas expression levels remained the same for all other conditions (Fig. 2b)^26^. We then investigated the levels of surface markers associated with T-cell activation, an important role played by DCs in the immune system. Levels of HLADR, also known as MHCII, and CD80, a costimulatory molecule on DC surfaces, showed no significant changes under all conditions, indicating that neither RPM nor matrix density had a significant effect on these markers (Fig. 2b). On the other hand, CD86, another costimulatory molecule on the surface of DCs, was significantly upregulated in static dense matrices compared to loose matrices (Fig. 2b). Under RPM conditions, there was a significant reduction in CD86 in dense matrices compared to controls (Fig. 2b). These data indicate that iDCs cultured statically have levels of surface markers that are either constant or higher in dense matrices than in loose matrices. This suggests that a denser, or normal, microenvironment results in more differentiated and proinflammatory cells than those that are cultured in a looser or aged matrix. Our results are in alignment with a study that showed that DCs cultured on higher stiffness 2D PDMS hydrogels showed increased activation^27^. Upon exposing these samples to accelerated aging onboard the RPM, we see that the levels of surface markers are either significantly reduced or remain constant. These data are in line with studies showing that aged individuals have a lower ability for DC differentiation^28,29^.

Cytokines secreted by DCs are also critical in activating T cells of the adaptive immune response. To investigate the effects of RPM conditioning and matrix density on cytokine release by iDCs, we collected supernatants from cells differentiated into iDCs under static or RPM culture in loose and dense matrix conditions for 3 days. Data indicating raw values of the concentration of cytokines secreted by iDCs can be found in Supplementary Fig. 2. As seen in the heatmap in Fig. 2c, there is an overall trend of increased concentration of cytokine secretion in dense matrices compared to loose matrices in statically cultured conditions, with a significant increase in the levels of CXCL8 and TNFα. CXCL8 is a DC activation marker, while TNFα induces T-cell differentiation^30,31^. This overall increase in cytokine secretion in dense matrices corroborates the higher expression of proinflammatory surface markers in dense matrices. When cultured on RPM, cytokine secretion is lowered from iDCs cultured in both loose and dense matrices compared to static controls (Fig. 2c). Specifically, we observed significant decreases in the concentrations of CXCL10, CCL2, IL-17A, IL-6, IFNγ, and TNFα from iDCs in dense matrices (Fig. 2c). CXCL10 is a crucial chemokine involved in the recruitment of T cells to the site of infections, whereas CCL2 plays a role in DC maturation^32,33^. IL-17A, IFNγ, and TNFα are involved in polarizing T cells of the adaptive immune response, and IL-6 plays an important role in regulating DC differentiation^34–37^. Data have shown that elderly individuals had lower concentrations of major cytokines such as CCL2, TNFα, CXCL8, IL1b, and IL-6 than their younger counterparts, which is in agreement with the lower concentration of cytokines secreted by samples exposed to RPM conditions^38^. This lower concentration of critical cytokines released under RPM conditions is also indicative of attenuation in the immunogenicity of monocyte-derived iDCs. Hence, we can imply from proinflammatory surface marker expression and cytokine secretion data that bioreactor-aged monocytes have reduced differentiation capabilities into iDCs.

### iDC antigen uptake is enhanced in dense matrices and independent of accelerated aging

**Fig. 3:**
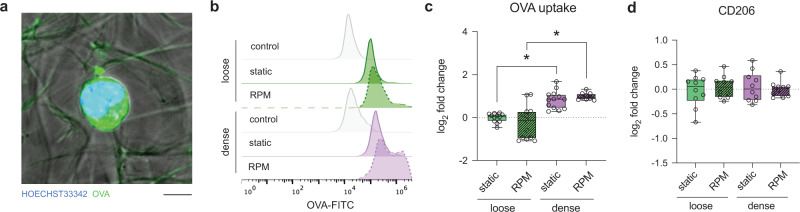
Quantitative analysis of OVA uptake by iDCs. Monocyte-derived iDCs were cultured for 3 days under static or RPM conditions in loose and dense matrices. FITC-labeled OVA was added to iDCs under their respective conditions for 1 h. **a** Representative image of iDCs cultured in a dense matrix under RPM conditions after 1 h of incubation with FITC-OVA. The scale bar represents 5 µm. **b** Representative histogram of fluorescence intensity of samples cultured under the different conditions following a 1-h incubation with FITC-OVA analyzed using flow cytometry. Control samples were cultured in the relative conditions with no FITC-OVA added. **c** Uptake of FITC-OVA calculated as the log_2_-fold change in geometric mean fluorescence intensity (gMFI) using flow cytometry. **d** Log_2_-fold change analysis of surface markers associated with uptake, namely, CD206, using flow cytometry. Log_2_-fold change was calculated relative to samples cultured in loose matrices under static conditions. Experiments were performed with at least four replicates. The box and whiskers graphs used have the center line at the median value. The upper and lower bounds of the box extend from the 25th to 75th percentiles, and the whiskers are plotted to the minimum and maximum values. * indicates significance at *p* ≤ 0.05.

Given that the primary role of iDCs is to scan and engulf antigens, we next investigated this specific cellular function using FITC-labeled ovalbumin (FITC-OVA) and quantified the amount of uptake by iDCs using flow cytometry. Figure 3a shows a representative image of iDCs after incubation with FITC-OVA for 1 h, indicating the uptake of FITC-OVA by iDCs. Additionally, representative histogram plots from flow cytometry confirmed the uptake of FITC-OVA in all conditions (Fig. 3b). Quantifying these data, we found that there was a significant increase in FITC-OVA uptake in iDCs cultured in dense matrices compared to those in loose matrices in both static culture and on the RPM (Fig. 3c). OVA uptake is mediated by macropinocytosis and clathrin-mediated endocytosis via the macrophage mannose receptor (MMR or CD206), which was found to be expressed similarly in all culture conditions (Fig. 3d)^39^. A possible reason for the difference in FITC-OVA uptake in loose and dense matrices might arise from CD206 activity and turnover rather than expression, highlighting the mechanoregulation of the iDC function through matrix porosity. In addition, flow cytometry data revealed no significant influence of matrix porosity on FITC-OVA uptake under static culture compared to RPM conditions. These data suggest that the accelerated aging of iDCs via RPM conditioning does not significantly decrease the capability of OVA uptake. These data are in agreement with previous findings showing that aged iDCs (RPM condition in our case) could efficiently internalize ovalbumin when compared to their younger counterpart^40^.

### A dense matrix attenuates iDC transcriptome changes under RPM conditions

**Fig. 4:**
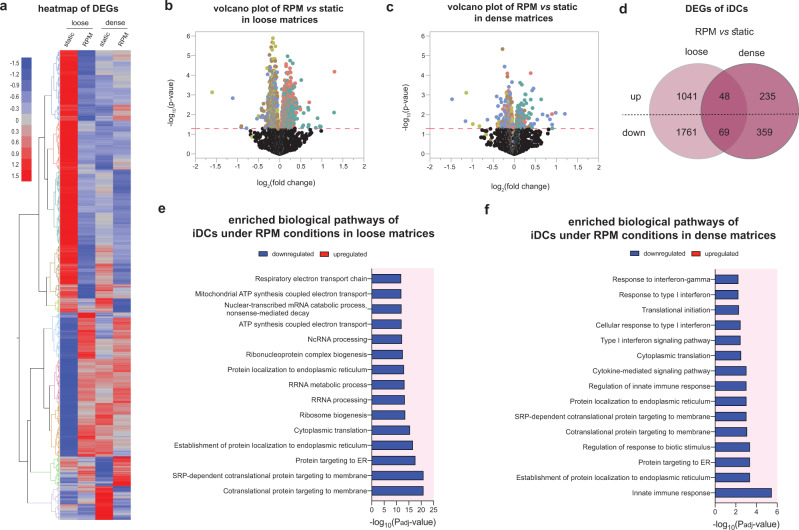
Transcriptome analysis of iDCs using RNA sequencing. iDCs were cultured for 3 days under static or RPM conditions in loose and dense matrices. **a** Heatmap of total DEGs in samples after RNA sequencing analysis. Volcano plots depicting up- and downregulated DEGs of iDCs cultured on the RPM compared to static in **b** loose and **c** dense matrices. **d** Venn diagram of the number of DEGs of iDCs cultured on the RPM compared to static in either loose or dense matrices. Top 15 enriched biological process pathways under RPM conditions in **e** loose and **f** dense matrices.

To date, few studies have investigated the effects of RPM exposure on the transcriptome profile of DCs cultured in 3D matrices. To further delve into the effects seen in the previous sections, we performed RNA sequencing on THP-1-derived iDCs cultured for 3 days statically or on the RPM in both loose and dense matrices. We began by analyzing the overall differentially expressed genes (DEGs) in the various cell culture conditions. Figure 4a shows a heatmap with the up- and downregulated DEGs in iDCs cultured in loose and dense matrices under static and RPM conditions. Volcano plots representing the up- and downregulated DEGs of RPM-conditioned samples compared to static controls in loose and dense matrices are shown in Fig. 4b, c. We observed that from iDCs cultured in loose matrices, there were 1089 differentially upregulated and 1830 differentially downregulated genes in samples cultured on the RPM compared to their static counterparts (Fig. 4d). However, there were only 283 differentially upregulated and 428 differentially downregulated genes in dense matrices despite being cultured on the RPM (Fig. 4d). Of these, there were 48 upregulated and 69 downregulated genes that were specifically altered in iDCs by being on the RPM and were independent of the matrix density that they were cultured in (Supplementary Fig. 3). We next analyzed the top 15 significant biological process pathways that were altered by RPM conditioning and different matrix densities. Figure 4e shows that in looser matrices, there is a downregulation of pathways primarily involved in cellular metabolism and protein localization. When antigens are engulfed by DCs, they are broken down and processed before being presented on the surface through the use of vesicles^41^. Although we observed no difference in the levels of antigen uptake between static and RPM conditions in the loose matrices described above (Fig. 3), a diminished ability to process and localize proteins could result in a decrease in the ability of DCs to present antigens on their surface, which may in turn lead to dysregulated immune responses by the adaptive immune system^42^. In fact, it has been reported that aging attenuates the antigen presentation ability of DCs^43^. On the other hand, in dense matrices, we observed that the pathways downregulated by RPM exposure compared to static controls were similarly involved in protein localization but also in the innate immune response and type I interferon responses (Fig. 4f). Interferon responses are critical for DCs to function optimally to mount effective T-cell responses^35^. It is essential to note that the significance of downregulation of the pathways in denser matrices is lower (a decrease of one order of magnitude) than that in loose matrices. This, along with the lower number of DEGs in Fig. 4d, alludes to the fact that iDCs cultured in higher-density matrices are less affected by RPM conditioning at the transcriptome level. Interestingly, our previous results showed that in a 3D collagen matrix (2 mg/mL), the transcriptome of T cells was less altered compared to samples cultured in 2D cell culture conditions^18^. In addition, it indicates that RPM conditioning, or accelerated aging, has more prominent effects on samples cultured in loose or aged matrices compared to dense, normal matrices.

### Mature DCs

Activated iDCs further develop and mature into mDCs, express elevated levels of HLADR, CD80, and CD86, and secrete more cytokines to effectively mount a T-cell response^22^. To ensure that our differentiation protocol effectively produced mDCs, we compared the levels of surface markers on mDCs to those expressed by iDCs under static culture. As shown in Supplementary Fig. 4a, in both loose and dense matrices, mDCs expressed significantly higher levels of CD11c, HLADR, CD80, and CD86 than iDCs, indicating an increase in DC maturation and activation. Supplementary Figure 4b shows a heatmap of the fold change in cytokine concentrations secreted by mDCs compared to iDCs cultured in loose matrices. Corresponding raw fold change data for all samples can be found in Supplementary Fig. 5. As expected, cytokine analysis revealed increased levels of secretion by mDCs compared to iDCs in both loose and dense matrices (Supplementary Fig. 4b). In addition, we analyzed changes in the transcriptome of mDCs compared to iDCs using RNA sequencing in both cell culture matrices. Supplementary Figure 4c shows a heatmap of the overall DEGs in mDC and iDC samples cultured statically in loose and dense tissue culture conditions. Volcano plots depicting the up- and downregulated DEGs in mDCs compared to iDCs in loose and dense matrices are shown in Supplementary Fig. 4d, e. Analysis revealed that in loose matrices, there were 2969 upregulated DEGs and 3454 downregulated DEGs, while in dense matrices, there were 2408 upregulated and 2573 downregulated DEGs (Supplementary Fig. 4f). Further analysis of the top 15 enriched biological process pathways altered by the differentiation process revealed that in both loose and dense tissue culture matrices, mDCs had significantly upregulated pathways that were primarily involved in the immune response, regulation of immune cytokine secretion, responses to cytokines, and immune-related signaling pathways (Supplementary Fig. 4g, h). These data confirm that mDCs are more mature and activated than iDCs and express the expected higher levels of surface markers and cytokines, as well as upregulated genes involved in immune response pathways.

### Cell surface markers, cytokine secretion, and mDC function altered by matrix porosity and RPM conditioning

**Fig. 5:**
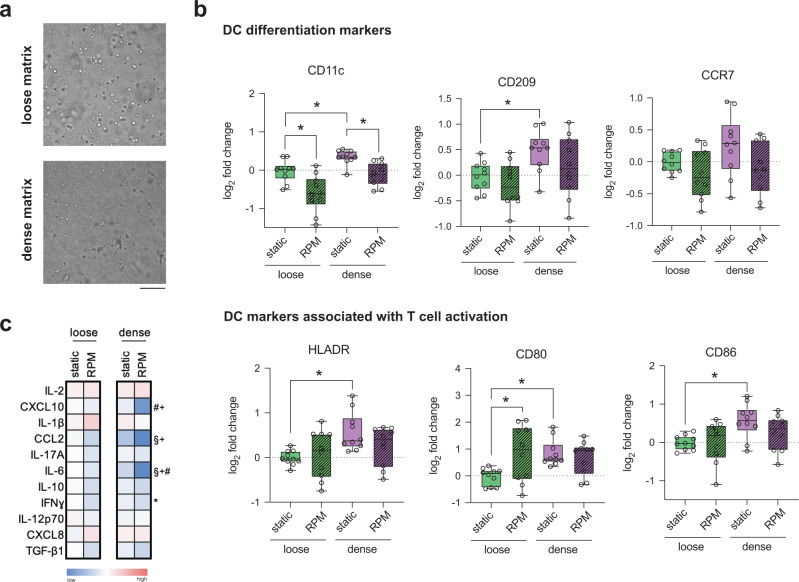
Quantitative analysis of mDC surface markers and cytokines. mDCs were cultured for 3 days under static or RPM conditions in loose and dense matrices. **a** Representative brightfield images of mDCs cultured in loose and dense matrices under static conditions. The scale bar represents 100 µm. **b** Analysis of surface markers associated with differentiation, namely, CD11c, CD209, and CCR7, and markers associated with T-cell activation, namely, HLADR, CD80, and CD86, using flow cytometry. The log_2_-fold change in geometric mean fluorescence intensity (gMFI) was calculated relative to samples cultured in loose matrices under static conditions. **c** Heatmap of the log_2_-fold change in cytokine secretion by mDCs cultured in loose and dense matrices under static and RPM conditions. The log_2_-fold change in the median concentration of cytokines (pg/mL) was calculated relative to cytokine secretion by mDCs cultured in loose matrices under static conditions. Experiments were performed with at least four replicates. The box and whiskers graphs used have the center line at the median value. The upper and lower bounds of the box extend from the 25th to 75th percentiles, and the whiskers are plotted to the minimum and maximum values. * indicates significance at *p* ≤ 0.05. For the heatmap, *indicates significant *p* ≤ 0.05 of samples in loose matrices cultured on the RPM compared to loose matrices under static conditions. # indicates significant *p* ≤ 0.05 of samples in dense matrices cultured on the RPM compared to dense matrices under static conditions. § indicates significant *p* ≤ 0.05 of samples in dense matrices cultured under static conditions compared to loose matrices cultured under static conditions. + indicates significant *p* ≤ 0.05 of samples cultured in dense matrices on the RPM compared to loose matrices on the RPM.

To determine how the same exact cell culture conditions subjected to differentiation of monocytes into iDCs affect the subsequent maturation of DCs, we cultured monocytes with a cocktail of GM-CSF, IL-4, TNFα, and ionomycin for 3 days statically and on the RPM to simulate accelerated aging. Likewise, we first analyzed morphological differences in mDCs cultured in loose and dense matrices under static conditions and on the RPM. Figure 5a shows that in loose matrices under static conditions, mDCs appear to be more round in shape than those cultured in dense matrices. mDCs cultured in dense matrices are larger in size and have dendrites that protrude from the cell (Fig. 5a). mDCs cultured in loose and dense matrices on the RPM had no morphological differences compared to those cultured statically. Next, we analyzed changes in surface marker expression levels associated with differentiation. A representative gating strategy for these markers is found in Supplementary Fig. 6. We found that mDC expression of CD11c was upregulated in dense matrices compared to loose matrices in static culture (Fig. 5b). In addition, the levels of CD11c in mDCs in both loose and dense matrices were significantly downregulated when RPM was conditioned compared to their respective static controls (Fig. 5b). Levels of CD209 were significantly increased in dense compared to loose matrices in static culture and remained constant in all other conditions, whereas CCR7 levels remained constant under any culture condition (Fig. 5b). Regarding surface markers associated with T-cell activation, the levels of HLADR, CD80, and CD86 in mDCs were significantly higher in dense matrices than in loose matrices under static conditions (Fig. 5b). When cultured on RPM, however, the expression levels remained unchanged except for CD80 (Fig. 5b). Interestingly, in loose matrices, mDC expression of CD80 was significantly higher when RPM conditioned compared to static controls (Fig. 5b). The increase in mDC surface marker levels cultured in denser matrices is in line with the results found in monocyte-derived iDCs seen above and further enforces the suggestion that the immunogenicity of DCs is increased in denser matrices. These data also point to the notion that accelerated aging, or RPM conditioning, does not alter the surface markers of mDCs as effectively as those seen in iDCs above.

We also investigated the effects of simulated aging via RPM conditioning and matrix density on cytokine secretion by mDCs. Raw fold change data values of the concentration of cytokines can be found in Supplementary Fig. 7. As seen in the heatmap in Fig. 5c, there appears to be decreased secretion of cytokines in samples cultured on board the RPM compared to static controls. CXCL10 secretion by mDCs is significantly lowered in dense matrices when cultured onboard RPM (Fig. 5c). mDC CCL2 and IL-6 secretion levels were significantly lower in denser matrices when cultured statically and onboard the RPM (Fig. 5c). However, IL-6 is also significantly lower in dense matrices cultured on the RPM compared to static controls. IFNγ was also found to be significantly lower in samples cultured on the RPM compared to static conditions in loose matrices (Fig. 5c). These data show that while RPM conditioning may not play a significant role in attenuating the levels of surface markers on the surface of mDCs, it significantly reduces the secreted concentration of specific key cytokines.

### T-cell activation by mDCs is altered by matrix density and RPM conditioning

**Fig. 6:**
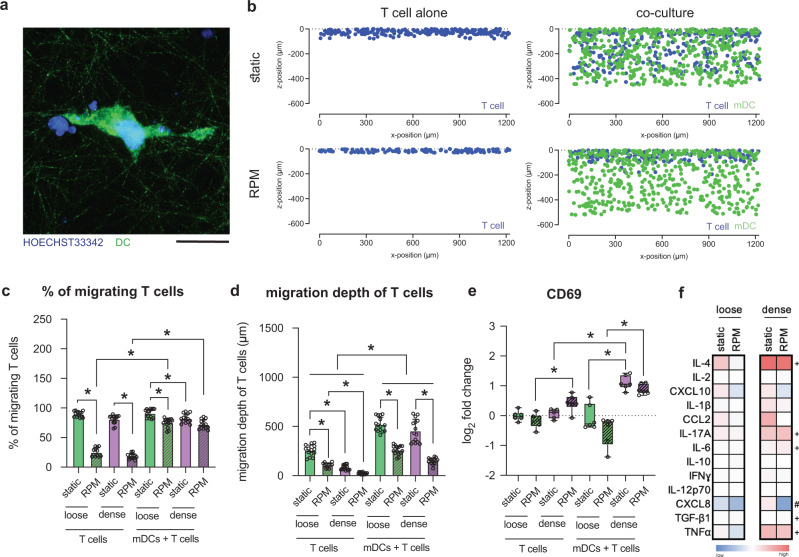
Coculture analysis of mDCs and T cells. mDCs were cocultured with T cells for 3 days in loose and dense matrices under static or RPM conditions. **a** Representative image of mDCs (green) interacting with T cells (blue) in a dense matrix under static conditions. The scale bar represents 25 µm. **b** Representative cross-section of mDCs (green) and T cells (blue) migrating into a dense matrix under static and RPM conditions. **c** Percent of migrating T cells, cultured alone or with mDCs, into loose and dense matrices under static or RPM conditions. **d** Migration depth (µm) of T cells cultured alone or with mDCs into loose and dense matrices under static or RPM conditions. **e** Analysis of CD69, a surface marker associated with T-cell activation, using flow cytometry. The log_2_-fold change in gMFI was calculated relative to the respective samples cultured in loose matrices under static conditions. **f** Heatmap of the log_2_-fold change in cytokine concentration secreted by mDCs in coculture with T cells cultured in loose and dense matrices under static and RPM conditions. The log_2_-fold change in the median concentration of cytokines (pg/mL) was calculated relative to the cytokine concentration of samples cultured in loose matrices under static conditions. Experiments were performed with at least four replicates. Bar graph data are shown as the mean ± SD. The box and whiskers graph used has the center line at the median value. The upper and lower bounds of the box extend from the 25th to 75th percentiles, and the whiskers are plotted to the minimum and maximum values. * indicates significance at *p* ≤ 0.05. For the heatmap, # indicates significant *p* ≤ 0.05 of samples in dense matrices cultured on the RPM compared to dense matrices under static conditions. + indicates significant *p* ≤ 0.05 of samples cultured in dense matrices on the RPM compared to loose matrices on the RPM.

The primary role of mDCs is to mount an effective adaptive immune response by activating T-cells, and they do this by forming an immune synapse and releasing cytokines^44,45^. This synaptic physical cell-to-cell interaction between the two cells is a critical step in effectively activating T cells by providing them with their necessary signal 1 and signal 2^46^. We thus investigated this pivotal immunological functional process of mDCs under the effects of RPM conditioning and matrix density. Through image quantification, we studied the infiltration of T cells into the loose and dense matrices when cultured statically or onboard the RPM. Figure 6a shows a representative image of an mDC-T-cell interaction in dense matrices when cultured statically. Figure 6b depicts the spatial distribution of mDCs and infiltrating T cells into dense tissue matrices during static culture and on the RPM as imaged and sectioned cross-sectionally. Quantitative analysis revealed that when T cells were cultured alone, there was a significantly lower percentage of migrating cells in RPM-conditioned samples (Fig. 6c). The infiltration of T cells was rescued when T cells were cocultured with mDCs, as measured by a higher percentage of T cells migrating into the collagen matrices (Fig. 6c). Potentially, this could be due to the secretion of CCL19 by DCs and due to chemokinesis, heightening the search function of T cells for HLADR on the surface of mDCs^47^. On board the RPM, however, the % of migrating T cells into matrices when cocultured with mDCs is still lower, significantly only for loose matrices. Under static culture conditions, denser matrices resulted in a significantly lower percentage of migrating T cells. In addition, T cells migrated less into both loose and dense matrices when cocultured with mDCs onboard the RPM compared to static controls (Fig. 6c).

As our mDCs were spatially distributed uniformly throughout the collagen matrices, we further quantified the migration depth of T cells. Overall, T cells migrated significantly deeper into the matrices when cocultured with mDCs than when mDCs were absent (Fig. 6d). We also observed that this infiltration into the matrices was significantly less when cultured onboard RPMs, regardless of whether they were cultured with mDCs or alone (Fig. 6d). In addition, the shorter migration distances of T cells in denser matrices are likely due to the differences in pore sizes (Fig. 6d). It is evident that T cells migrate further into the matrices in the presence of mDCs perhaps because mDCs “call” the T cells to them by the release of CCL19 and formation of their gradients^48–50^. However, when conditioned using RPM, we hypothesize that there may be a lack of gradient formed by the chemokines released by mDCs; therefore, T cells are unable to effectively locate and synapse with mDCs. However, the chemotaxis of T cells toward CCL19 gradients is still a controversial topic^51^.

For T cells that synapse with mDCs, we analyzed their activation under various culture conditions by measuring the expression of CD69, an early T-cell activation marker^52^. A representative gating strategy for this marker is found in Supplementary Figure 8. T cells alone exhibited no change in CD69 expression when cultured statically in any of the matrices (Fig. 6e). As expected, there were higher levels of CD69 on T cells cocultured with mDCs than on T cells cultured alone (Fig. 6e). On board the RPM, however, there was a significant increase in CD69 levels in T cells cultured alone in denser matrices (Fig. 6e). This is also in line with the higher activation and maturation of iDCs and mDCs in denser matrix conditions, as seen in the data above. When cocultured with mDCs, we observed significantly higher expression levels of CD69 in T cells in denser matrices for both statically cultured samples and those cultured on the RPM (Fig. 6e). Again, this is expected due to the higher activation levels of mDCs cultured in denser matrices. Furthermore, we analyzed the cytokine secretion profiles of mDCs when cocultured with T cells under various cell conditions. Figure 6f shows a heatmap representation of the cytokine concentration fold change compared to concentrations obtained from mDCs cultured in loose matrices statically. Raw concentration data of cytokines can be found in Supplementary Fig. 9. As expected, we observed elevated cytokine concentrations in cells cultured in denser matrices (Fig. 6f). In denser matrices cultured onboard the RPM, there are significantly lower levels of CXCL8, a potent chemotactic factor for a variety of immune cells, including T cells, than in their static counterparts^53^. When RPM conditioned, concentrations of TGF-β1 involved in T-cell function^54^; IL-6, a T-cell survival factor^55^; IL-17A, involved in polarizing T cells^34^; IL-4, which influences T-cell fate^56^; and TNFα, which promotes the activation and proliferation of T cells^57^, are higher in denser matrices relative to the concentration secreted by mDCs cultured in loose matrices onboard the RPM (Fig. 6f). These data support previous reports that show higher levels of cytokine concentration secreted by DCs from younger individuals compared to aged individuals^38^. The lack of change in cytokine secretion by mDCs cultured onboard RPM compared to static controls is in line with the activation levels of T cells that also had no significant differences in these same conditions (Fig. 6e).

### The effects of RPM conditioning on the mDC transcriptome are attenuated in dense matrices

**Fig. 7:**
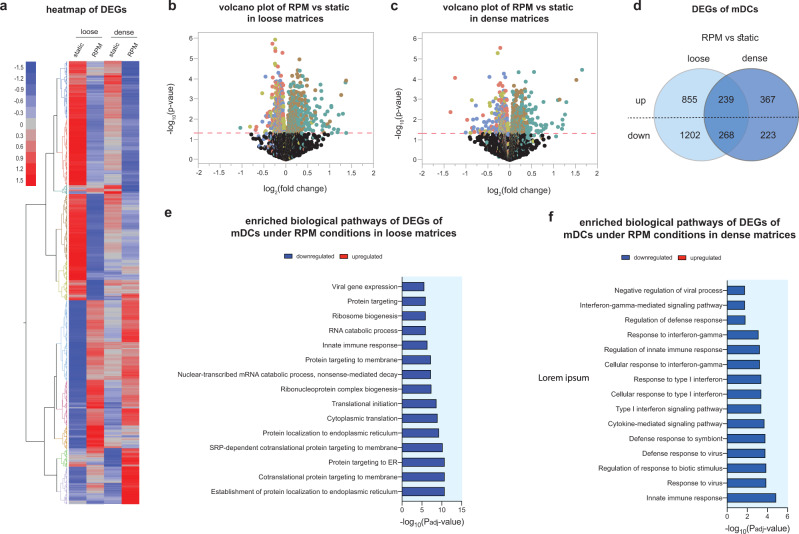
Transcriptome analysis of mDCs using RNA sequencing. mDCs were cultured for 3 days under static or RPM conditions in loose and dense matrices. **a** Heatmap of total DEGs in samples after RNA sequencing analysis. Volcano plots depicting up- and downregulated DEGs of mDCs cultured on the RPM compared to static in **b** loose and **c** dense matrices. **d** Venn diagram of the number of DEGs of mDCs cultured on the RPM compared to static in either loose or dense matrices. Top 15 enriched biological process pathways under RPM conditions in **e** loose and **f** dense matrices.

To investigate further, we performed RNA sequencing on monocyte-derived mDCs cultured for 3 days statically or on board RPM and in both loose and dense matrices. We began by analyzing the overall DEGs in various conditions. Figure 7a shows a heatmap with the up- and downregulated DEGs in mDCs cultured under the various conditions. Volcano plots representing the up- and downregulated DEGs under RPM conditions compared to static controls in loose and dense matrices are shown in Fig. 7b, c. In loose matrices, we can see that there are 1094 differentially upregulated and 1709 differentially downregulated genes in samples conditioned by RPM compared to static controls (Fig. 7d). In dense matrices, however, there were only 606 differentially upregulated and 491 differentially downregulated genes (Fig. 7d). Of these, there were 239 upregulated and 268 downregulated genes that were specifically altered in mDCs by being cultured onboard the RPM and were independent of the matrix density that they were cultured in (Supplementary Fig. 10). We next analyzed the top 15 significant biological process pathways that were altered by exposure to RPM conditioning and different matrix densities. Figure 7e shows that in loose matrices, there is a downregulation of pathways primarily involved in protein targeting and localization as well as the innate immune response. On the other hand, in dense matrices, we observed that more pathways related to immune and interferon responses were downregulated in samples cultured on RPM than in static controls (Fig. 7f). Interferon responses are critical for DCs to function optimally and mount effective T-cell responses^35^. However, as noticed with the iDC transcriptome pathways, it is important to note that the significance of downregulation of the pathways in mDCs in dense matrices is lower than those in less dense matrices (~ one order of magnitude less). This, along with the lower number of DEGs that are altered in Fig. 7d, similarly alludes to the fact that mDCs cultured in higher-density matrices are less affected by RPM conditions at a transcriptome level.

## Discussion

**Fig. 8:**
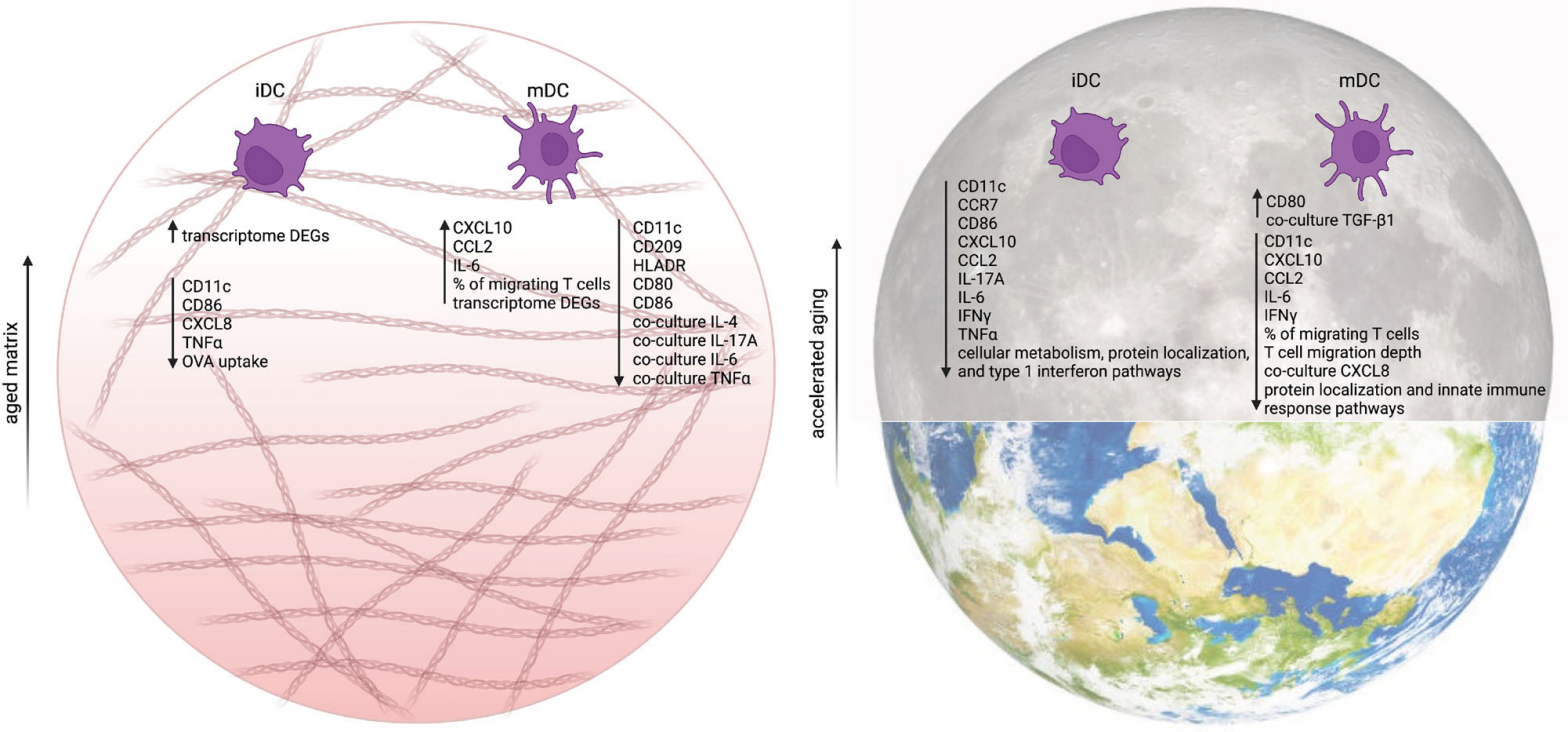
Summary of the effects of matrix density and RPM conditioning on monocyte-derived iDCs and mDCs. Looser matrix density recapitulates aged tissue, and a summary of the effects on iDCs and mDCs due to this, compared to dense matrix controls, is shown in the panel on the left. Exposure to simulated microgravity mimics the effects of accelerated aging, and a summary of these effects of RPM conditioning, compared to static controls on Earth, on iDCs and mDCs is shown in the panel on the right. This figure was created with BioRender.com.

It is well acknowledged that the immune system is adversely affected by aging in similar ways as it is affected by exposure to microgravity^3,6,58^. Thus, simulating the effects of microgravity on cells is becoming an increasingly useful method for recapitulating and accelerating the effects of aging. However, due to the complexity and ethical concerns of obtaining samples, we are unable to study the effects of microgravity on cells residing within human tissue. We recently showed that 3D biomimetic models could replicate the native microenvironment of tissue-resident immune cells, and this approach replaces the need for animal models or invasive cell retrieval from donors^17,18,59^. In this work, we focus on the differentiation and maturation of aged DCs conditioned using sµG via an RPM bioreactor and cultured within loose and dense 3D matrices, which we use as analogs for aged and normal tissues. A summary illustration of our findings can be seen in Fig. 8. Our data imply that a denser tissue microenvironment alone, with inherently smaller pore sizes, increases the overall immunogenicity potential of both iDCs and mDCs, as seen by an increase in surface marker expression and cytokine secretion (Fig. 2 and Fig. 5). Upon conditioning by RPM, we observed a decrease in specific surface markers as well as secreted cytokines by both iDCs and mDCs (Fig. 2 and Fig. 5). While our results show a decrease in most DC secreted cytokines upon exposure to RPM conditioning as well as a looser tissue niche, both of which simulate “aged” conditions, studies of cytokine levels in astronauts’ blood has shown dysregulation in certain cytokines, such as an increase in TNFα, IL-10, and IL-6 levels, during spaceflight^60–63^. These differences could be due to the fact that our system isolates and focuses on only DC differentiation and function. Furthermore, our DCs are cultured in 3D matrices, replicating tissue resident conditions, whereas cytokine levels from astronauts are obtained from peripheral blood and consist of multiple cell types. Bearing in mind the immune response of these peripheral blood cells is also modulated via crosstalk from other organ systems.

Interestingly, at the transcriptome level, we observed that a younger tissue niche, a denser collagen-formulated microenvironment, better “protected” the cells from the effects of RPM conditioning compared to a looser, more aged tissue niche. This is in line with our previous results that showed attenuated effects of simulated microgravity on the transcriptome profile of T cells cultured in 3D microenvironments versus 2D cell culture dishes^18^. In that study, using computational simulations, we have further shown that shear forces within collagen matrices on the RPM are negligible and do not add to the effects seen under simulated microgravity^18^. Therefore, the altered immune potency, seen through RPM conditioning here, is due to the cancellation of gravitational forces alone.

In healthy immune systems, iDCs uptake and present antigens to cells of the adaptive immune system. Interestingly, we observed no significant changes in their ability to take up OVA when cultured onboard the RPM (Fig. 3). RNA sequencing data indicated the downregulation of pathways involved in vesicle and protein localization (Fig. 4). This could imply that while uptake of antigen by iDCs is not affected by RPM conditioning, the subsequent processing steps involved in presenting the antigen to T cells could be attenuated, which would, in turn, affect the overall immune response.

Notably, we observed fewer overall changes in mDCs at the protein and transcriptome levels than in iDCs, even in denser biomimetic microenvironments. We hypothetically attribute this to mDCs being more mature than iDCs after 3 days of culture and, therefore, less “plastic” in terms of their immune potency being altered by external factors when they reach the lymph nodes for T-cell interactions. To reiterate, DCs are mechanosensitive to their biophysical microenvironment. For example, the immune profile of mouse DCs migrating from peripheral tissues to lymph nodes has been found to be biophysically altered by elevated fluid shear forces and physical interaction with endothelial cells of the lymphatic vessels^64^. In addition, with accelerated aging via RPM, the migration depth of T cells into the matrices is significantly less, limiting T-cell interaction with mDCs. This could also affect our immune responses and should be further addressed in future studies that focus on migration dynamics. Of the T cells that do synapse with mDCs in this condition, their activation levels are also lowered. We have performed mechanobiology studies on T-cell interactions using artificial APCs, and by varying the stiffnesses of the “synapse” via polyacrylamide gels, we have found differential CD69 expression (unpublished data). It is, therefore, possible that mDCs cultured on the RPM have altered cytoskeleton dynamics, as reported for other cell types^65^, and this mechanically changed the actin-rich synapse of mDCs, affecting the CD69 activation levels on T cells^66^.

In summary, RPM conditioning of DCs cultured in biomimetic 3D collagen hydrogel matrices shows significant changes in surface marker expression, cytokine secretion, cellular function, and gene expression (Fig. 8). The majority of these changes signify a decrease in immunological potency, thus revealing that DC impairment has a role in age-related and space travel-related immune suppression. Following this, the mechanosensing mechanisms of DCs, pertaining to the lack of gravitational forces, should be explored to shed light on the causes of the reduced immune responses we reported here. We are possibly illuminating the way to improve the quality of life for the aged in our society on Earth and, de novo, countermeasures for astronauts participating in deeper and long-duration space missions from an immunological perspective. In addition, RPM conditioning can age in vitro cell culture and potentially organoid systems. Routinely, animal models are often used as a tool for such aged studies; therefore, our approach demonstrated here can be used as a replacement approach to reduce the need for in vivo studies involving aging.

## Methods

### Reconstitution of 3D collagen matrices, cell culture, monocyte-derived DC differentiation, and imaging

3D collagen matrices were prepared by mixing type I collagen from rat tail (Advanced BioMatrix, Inc. San Diego, CA, USA), 0.1% acetic acid (Sigma‒Aldrich, St. Louis, MO, USA), and 500 mM phosphate buffer (Sigma‒Aldrich, St. Louis, MO, USA) on ice at a concentration of either 1 mg/mL (loose matrix) or 3 mg/mL (dense matrix), as previously published^67^. The prepared collagen solution was transferred onto glutaraldehyde-coated coverslips (13 mm in diameter; VWR, Darmstadt, Germany) to allow for covalent binding of the collagen matrix via a lysine side chain^68^. Fibrillogenesis of the collagen matrix occurred by placing the coverslips at 37 °C, 5% CO_2_, and 95% humidity. Next, the 3D collagen matrices were washed 3 times with phosphate buffer saline (PBS; Thermo Fisher Scientific Inc., UK). Next, the 3D collagen matrices were placed in 4-well plates (Thermo Fisher Scientific Inc., Dreieich, Germany) and kept in PBS (Thermo Fisher Scientific Inc., UK) prior to use to prevent drying.

The human monocytic cell line THP-1 (AddexBio; cell line C0003024, USA) and the human T lymphocyte cell line Jurkat (AddexBio; cell line C0003039, USA) were maintained in RPMI-1640 cell culture medium supplemented with 10% fetal bovine serum (FBS), 1% HEPES, 1% sodium pyruvate, 0.01% beta-mercaptoethanol and 1% penicillin/streptomycin at 37 °C, 95% humidity and 5% CO_2_ (standard cell culture conditions). Cell culture medium and supplements were purchased from Gibco, Invitrogen, Thermo Fisher Scientific Inc., Dreieich, Germany.

For DC cell studies, 1 × 10^5^ THP-1 cells were seeded onto prepared matrices in FBS-free RPMI-1640 cell culture medium and placed in the incubator overnight to allow for infiltration of the cells into the collagen matrices. To induce the differentiation of THP-1 cells into DCs, the cells were cultured under standard cell culture conditions or on an RPM for 3 days in FBS-free RPMI-1640 cell culture medium supplemented with 200 ng/mL IL-4 and 50 ng/mL GM-CSF for iDC differentiation or in the presence of 200 ng/mL IL-4, 50 ng/mL GM-CSF, 20 ng/mL TNFα, and 200 ng/mL ionomycin for mDC differentiation. The differentiation protocol was adopted from Berges et al.^69^. All activating cytokines were purchased from Biolegend, USA. In addition, for the duration of the experiments, samples were cultured within custom-made PDMS microvessels as previously described^16^.

For representative images of cell culture matrices, loose and dense matrices were reconstituted and stained with 50 µM TAMRA and SE (5-(and-6)-carboxytetramethylrhodamine, succinimidyl ester; Thermo Fisher Scientific, USA) for 30 mins and washed 3 times with PBS (Thermo Fisher Scientific Inc., UK). Matrices were visualized using a STED superresolution microscope with a 63× water objective (LEICA, Germany). For representative imaging of iDCs and mDCs in loose and dense matrices, samples were cultured as stated above and imaged using a brightfield microscope (DMi8 S microscopy platform, Leica, Germany).

### Coculture of mDCs and T cells

For mDC and T-cell coculture analysis, mDCs were first differentiated as mentioned above in both 1 and 3 mg/mL collagen matrices under static conditions or on the RPM for 3 days. Next, samples were removed from their respective culture conditions, and PDMS microvessels and differentiation medium were removed. Samples were then washed 2 times with PBS (Thermo Fisher Scientific Inc., UK) and subsequently stained with 1 μM Cell Tracker™ CMFDA Green (Thermo Fisher Scientific, USA) for 10 min under standard cell culture conditions. Afterward, samples were washed three times with PBS (Thermo Fisher Scientific Inc., UK), and new microvessels were placed on the samples along with fresh FBS-free RPMI-1640 cell culture medium. For the coculture with T cells, 1 × 10^5^ Jurkat T cells were added before samples were placed back in their original conditions for an additional 3 days of culture. For the T-cell control, 1 × 10^5^ Jurkat cells were added to wells containing FBS-free RPMI-1640 cell culture medium in PDMS microvessels. Samples were then placed on the RPM or under static conditions in the same incubator.

### Setting of RPM

All RPM experiments were performed on a desktop RPM (Airbus Defense and Space Netherlands B.V., Leiden, The Netherlands). The RPM was placed inside an incubator with standard cell culture conditions (37 °C, 5% CO_2_, and 95% humidity). Afterward, 4-well cell culture plates (Thermo Fisher Scientific Inc., Dreieich, Germany) were placed at the center of rotation of the RPM to maintain the quality of sμG as suggested by the manufacturer of the RPM. The operational setting of the RPM was adjusted for a 3D random mode with random motion and random direction while maintaining an average velocity of 60 deg/s, as established^70^. Control samples under static conditions were placed in the same incubator next to the RPM.

### Quantitative analysis of cell surface markers

Collagen matrices were digested by incubation with 6 mg/mL collagenase (Gibco, Thermo Fisher Scientific Inc., Dreieich, Germany) solution prepared in RPMI-1640 medium for 10 min under standard cell culture conditions^59^. To quantify the expression of surface markers of iDCs and mDCs, cells were stained with antibodies against CD11c (Cat#: 337234), CCR7 (Cat#: 353206), CD80 (Cat#: 305236), CD86 (Cat#: 374214), HLADR (Cat#: 307636), CD209 (Cat#: 330106), and CD206 (Cat#: 321138) for 30 min on ice. All antibodies were diluted at a ratio of 1:250 in PBS (Thermo Fisher Scientific Inc., UK). For coculture cell surface marker analysis, mDCs and T-cells were stained using Zombie Violet Fixable Viability Kit (Cat#: 423114) at a dilution of 1:1000 in PBS (Thermo Fisher Scientific Inc., UK) as well as with an antibody against CD69 (Cat#: 310914), also diluted at a ratio of 1:250 in PBS (Thermo Fisher Scientific Inc., UK). All antibodies and the viability kit were purchased from Biolegend, USA. Clone, conjugated fluorochrome, isotype, and catalog number are listed in Supplementary Table 1. Afterward, stained cells were analyzed using the Cytek Aurora flow cytometer (4 L 16V-14B-10YG-8R) that utilizes an unmixing (compensation) program from the SpectroFlo software. Experiments were performed in at least 4 replicates. Analysis was then performed using FlowJo software (Becton, Dickinson and Company, NJ, USA).

### Cytokine quantification

To study the immune potency of the cells, the cell culture medium of differentiated iDCs and mDCs alone or differentiated mDCs cultured together with Jurkat T cells were collected, and the secretion of proinflammatory cytokines, namely, IL-4, IL-2, CXCL10, IL-1β, CCL2, IL-17A, IL-6, IL-10, IFNγ, IL-12p70, CXCL8, TNFα, and TGF-β1, was analyzed using bead-based ELISA (Biolegend, San Diego, CA, USA). Experiments were performed in at least 4 independent replicates.

### iDC uptake assay and imaging

iDCs were cultured, as mentioned above, in loose and dense collagen matrices either under static or RPM conditions. After 3 days of differentiation, 10 µg of OVA-FITC peptide was added for 1 h. Next, the supernatant was removed, and the samples were washed two times with PBS (Thermo Fisher Scientific Inc., UK). Next, samples were analyzed for FITC signal using the Cytek Aurora flow cytometer (4 L 16V-14B-10YG-8R) that utilizes an unmixing (compensation) program from the SpectroFlo software. Experiments were performed in at least four replicates. Analysis was then performed using FlowJo software (Becton, Dickinson and Company, NJ, USA). Additional samples were also stained with HOECHST 33342 (dilution 1:10,000 in PBS; Invitrogen, Carlsbad, CA, USA) and imaged using a STED superresolution microscope (LEICA, Germany) to obtain representative images of iDC cells that had taken up OVA-FITC peptide.

### Image analysis and quantification of infiltration of T cells

Prior to adding Jurkat T cells to the coculture, mDCs were stained with Cell Tracker™ CMFDA Green (Thermo Fisher Scientific, USA) for 10 min under standard cell culture conditions. Afterward, samples were washed three times with PBS (Thermo Fisher Scientific Inc., UK) before T cells were added and cultured for an additional 3 days. Next, cells were fixed with 4% paraformaldehyde (Biolegend, USA) for 10 min. Samples were then washed three times with PBS (Thermo Fisher Scientific Inc., UK). Afterward, samples were stained with Hoechst-33342 (dilution 1:10,000 in PBS; Invitrogen, Carlsbad, CA, USA) overnight before being washed three times with PBS (Thermo Fisher Scientific Inc., UK). Cell imaging was performed using a STED superresolution microscope with a 63× water objective (LEICA, Germany).

T-cell migration into collagen matrices was quantitatively determined by analyzing individual cell nuclei with only Hoechst-33342 fluorescence signal (and not Cell Tracker™ CMFDA Green), as previously reported^67^. *Z*-stack images with a spacing of 5 µm were obtained using an epi-fluorescence microscope with an automatic scanning stage (DMi8 S; Leica, Germany) using a ×10 dry objective (Leica, Germany). For each experiment, four different positions of each cell culture condition were analyzed. The *z*-position of cell nuclei as a function of migration distance was examined using a custom-built MATLAB script (MATLAB R2019b; MathWorks Inc., USA). Cells located >20 µm below the collagen matrix surface were classified and counted as migrated cells. The maximum migration distance was defined as the distance that was crossed by 10% of all migrating cells. The percentage of migrating T cells was determined as a percentage of the total number of T cells per field. Experiments were performed in at least three independent replicates.

### RNA isolation and purification

RNA was extracted using TRIzol (Invitrogen, Thermo Fisher Scientific, Inc., Dreieich, Germany), followed by chloroform extraction (Sigma‒Aldrich, Schnelldorf, Germany) using the manufacturer’s protocol. Afterward, purification using the RNeasy mini kit (Qiagen, Hilden, Germany) was performed according to the manufacturer’s protocol. The obtained RNA concentration and purity (the ratio of absorbance at 260 and 280 nm) were quantified using a Nanodrop (Thermo Fisher Scientific, Inc., Dreieich, Germany) and confirmed by a Qi RNA kit using a Qubit 4 fluorometer (Thermo Fisher Scientific, Inc., Dreieich, Germany) prior to performing RNA sequencing.

### RNA sequencing and analysis

Purified RNA samples were prepared with an NEB Ultra II RNA kit (New England Biolabs, Ipswich, MA, USA) according to the protocol instructions using the NEBNext Poly(A) mRNA Magnetic Isolation Module (New England Biolabs, Ipswich, MA, USA) and uniquely dual indexed. The resulting library concentration, size distribution, and quality were assessed on a Qubit 4 fluorometer (Thermo Fisher Scientific, Inc., Dreieich, Germany) with a dsDNA high sensitivity kit (Invitrogen, Carlsbad, CA, USA) and on a 4200 TapeStation using a High Sensitivity D5000 kit (Agilent, Santa Clara, CA, USA). Based on these results, libraries were normalized according to their molarity and pooled, and then quantified with a library quantification kit for Illumina platforms (Roche, Basel, Switzerland) on a StepOnePlus qPCR machine (Thermo Fisher Scientific, Inc., Dreieich, Germany). Finally, pooled libraries were loaded at 350 pM with 1% PhiX on S2 FlowCell and paired-end sequenced (2 × 150 bp) on a NovaSeq 6000 next-generation sequencer (Illumina, San Diego, CA, USA). RNA-Seq was performed in triplicate.

Raw FASTQ sequenced reads were first assessed for quality using FastQC v0.11.5 (available online at http://www.bioinformatics.babraham.ac.uk/projects/fastqc/)^71^. The reads were then passed through Trimmomatic v0.36^72^ for quality trimming and adapter sequence removal with the following parameters (ILLUMINACLIP: trimmo-matic_adapter.fa:2:30:10 TRAILING:3 LEADING:3 SLIDINGWINDOW:4:15 MINLEN:36). The surviving trimmed read pairs were then processed with Fastp^73^ to remove poly-G tails and Novaseq/Nextseq-specific artifacts. Following quality trimming, the reads were assessed again using FastQC. After QC and QT, the reads were aligned to the human reference genome GRCh38.p4 using HISAT2^74^ with the default parameters and by providing the –dta flag. The resulting SAM alignments were then converted to BAM format and coordinated sorted using SAMtools v1.3.1^75^. The sorted alignment files were then passed through HTSeq-count v0.6.1p1^76^ using the following options (-s no -t exon -I gene_id) for raw count generation. Concurrently, the sorted alignments were processed through Stringtie v1.3.0^77^ for transcriptome quantification. Briefly, the process was stringtie → stringtie merge (to create a merged transcriptome GTF file of all the samples) → stringtie (this time using the GTF generated by the previous merging step). Finally, Qualimap v2.2.2^78^ was used to generate RNA-Seq-specific QC metrics per sample.

RNA-Seq data were merged using the NASQAR toolbox (publicly accessible at http://nasqar.abudhabi.nyu.edu/; accessed on 20 March 2022)^79^. Raw counts of genes were first converted into TPM^80^. These data were then used with JMP Genomics (JMP®, Version <*9*>. SAS Institute Inc., Cary, NC, 1989–2021) software to determine differentially expressed genes (DEGs; FC ≥ 2, FDR ≤ 0.05). Idep96 (http://bioinformatics.sdstate.edu/idep96/; accessed on 17th June 2022) was used to determine the top enriched GO biological process pathways using the GAGE method (FDR ≤ 0.2)^81^.

### Data and statistical analysis

Statistical significance was determined using unpaired one-way ANOVA followed by the Kruskal‒Wallis post hoc test and Dunn’s test for multiple comparisons using Prism 9 (GraphPad Software Inc., San Diego, USA), and the level of significance was set to *p* ≤ 0.05. Unless otherwise stated, all experiments were performed in at least three replicates. Data are represented as the mean ± standard deviation.

### Reporting summary

Further information on research design is available in the Nature Research Reporting Summary linked to this article.

## Supplementary information

Supplementary Information

reporting summary

## Data Availability

The datasets generated and analyzed for this study can be accessed through Gene Expression Omnibus (GEO) under accession number GSE232153.

## Code availability

The custom codes used for this study can be provided upon request from the corresponding author.
