# Supplementary material for: Effects of an aged tissue niche on the immune potency of dendritic cells using simulated microgravity
Source: NPJ Aging. 2023 Jul 1;9(1):14. doi: 10.1038/s41514-023-00111-7 (PMC10314949; doi:10.1038/s41514-023-00111-7)
Supplement: Supplementary file 1 — Supplementary Information [file 41514_2023_111_MOESM1_ESM.pdf]

Supplementary Information

Effects of an aged tissue niche on the immune potency of dendritic cells using simulated microgravity

Mei ElGindi[1], Jiranuwat Sapudom[1], Anna Garcia Sabate[1], Brian Chesney Quartey[1], Aseel Alatoom[1], Mohamed Al-Sayegh[2], Rui Li[4], Weiqiang Chen[3,4], Jeremy Teo[1,3,4,*].

1. Laboratory for Immuno Bioengineering Research and Applications, Division of Engineering, New York University Abu Dhabi, Abu Dhabi POBox 129188, United Arab Emirates
2. Biology Division, New York University Abu Dhabi, P.O. Box 129188, Abu Dhabi, United Arab Emirates
3. Department of Mechanical and Aerospace Engineering, New York University, 6 MetroTech Center, Brooklyn, NY, 11201 USA
4. Department of Biomedical Engineering, New York University, 6 MetroTech Center, Brooklyn, NY, 11201 USA

*Correspondence: jeremy.teo@nyu.edu; Tel.: +971-2-6286689

Supplementary Table 1: Antibodies used for flow cytometry in this study

Marker	Fluorochrome	Host/Target	Isotype	Clone	Catalog Number
CD11c	PerCP	Mouse anti-Human	IgG1, κ	Bu15	337234
CCR7	Alexa Fluor 488	Mouse anti-Human	IgG2a, κ	G043H7	353206
HLADR	Brilliant Violet 421	Mouse anti-Human	IgG2a, κ	L243	307636
CD80	Brilliant Violet 711	Mouse anti-Human	IgG1, κ	2D10	305236
CD86	Brilliant Violet 605	Mouse anti-Human	IgG1, κ	BU63	374214
CD206	Brilliant Violet 510	Mouse anti-Human	IgG1, κ	15-2	321138
CD209	PE	Mouse anti-Human	IgG2a, κ	9E9A8	330106
CD69	APC/Cyanine7	Mouse anti-Human	IgG1, κ	FN50	310914

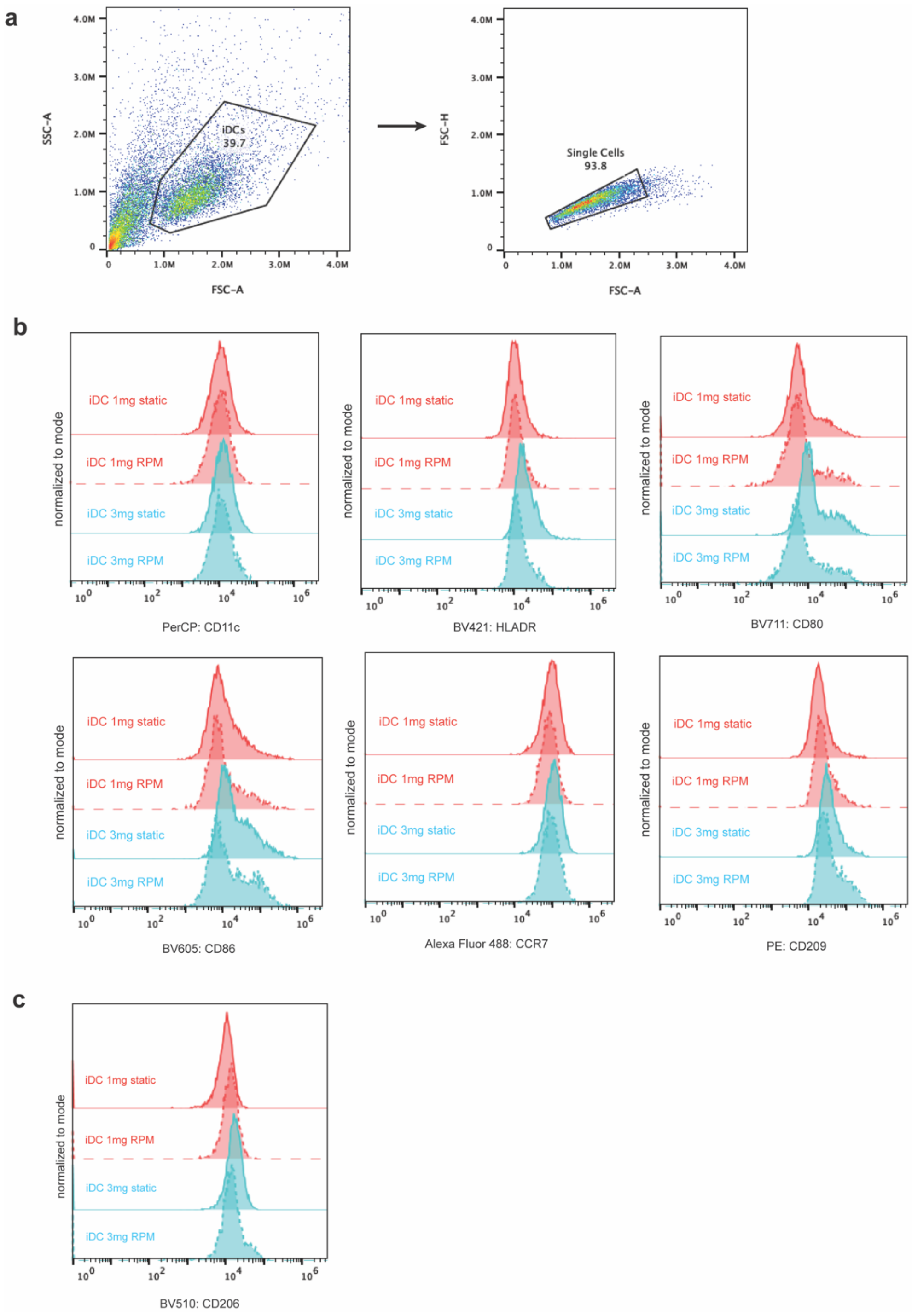

Supplementary Figure 1: a. Representative gating strategy for iDCs cultured under different conditions. Initial cell population was gated on FSC and SSC to remove cell debris and dead cells. The population was then gated to isolate single cells and remove any cell aggregates. This population was then used in the histogram analysis. **b.** Representative fluorescent histograms used in the study. This representation was used for the data analysis in Figure 2B in the manuscript. **c.** Representative fluorescent histogram of CD206 used in the study. This was used for the data analysis in Figure 3D in the manuscript.

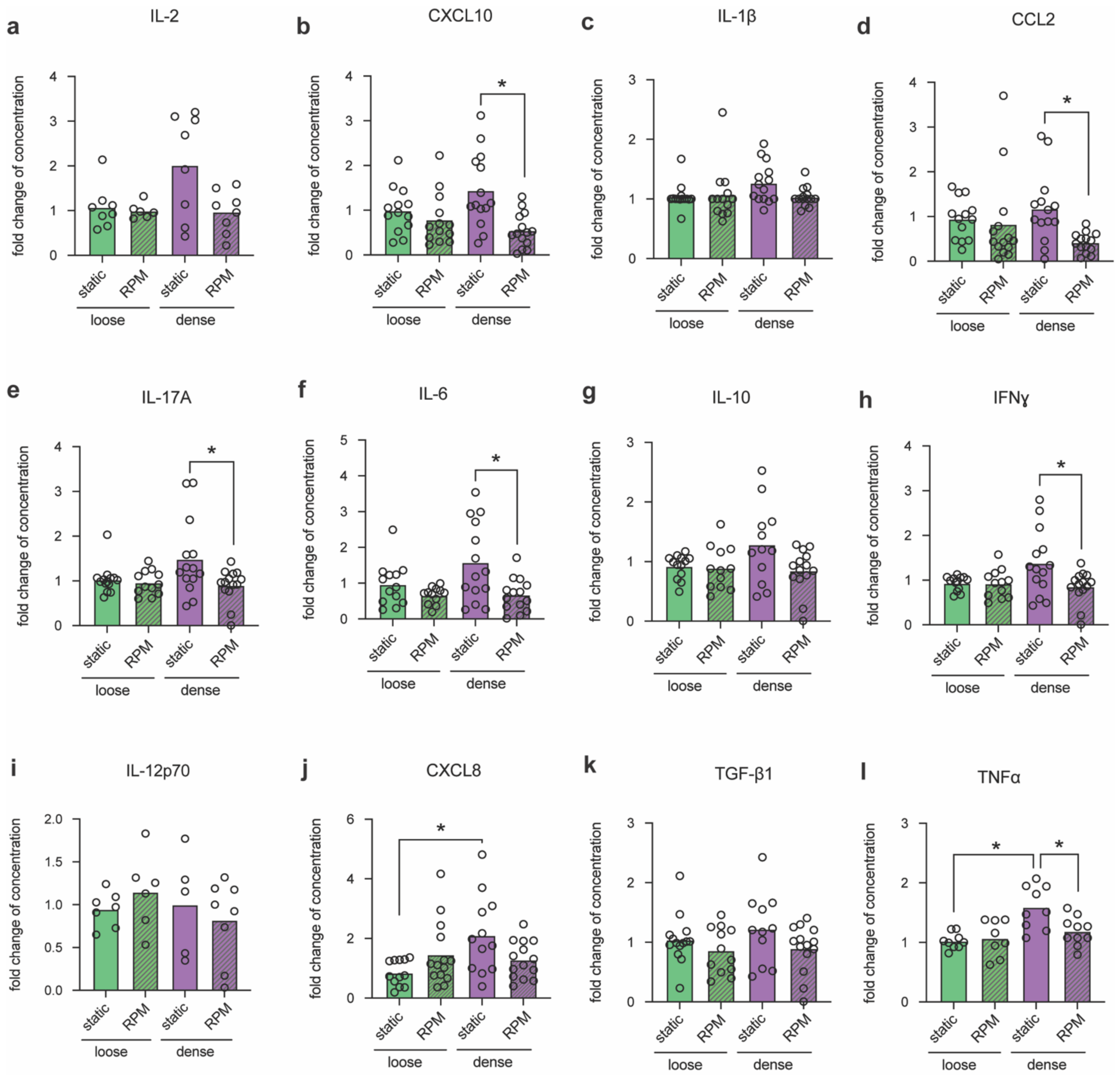

Supplementary Figure 2: Bar graph analysis of cytokine secretion by iDCs cultured for 3 days at static or RPM conditions in loose and dense matrices. Fold change of concentration of cytokine (pg/mL) calculated relative to samples cultured in loose matrices at static conditions.. Data are shown as mean +/- SD. * indicates significant $p \leq 0.05$.

up-regulated

ZNF740	C16orf54	F2RL3	SYNPO	ETS1
FLCN	NATD1	SLITRK2	ATXN2L	SFRP1
TNRC6C	ZMIZ2	C3	GPT2	NPTXR
NOL9	ARAP3	LINC00622	RUNX2	CYP1B1
RNF149	CLK4	CLK1	SLC1A4	MRC2
ZNF446	C19orf68	MMP14	KCNC3	ZFP92
SAFB	SLC45A4	SKIL	KLF16	CYP1B1-AS1
KIAA0930	GBGT1	C19orf80	SMAD7	MIR1286
CTSD	BRD4	MEGF8	SAMD1	
MPRIP	ITGAL	EPPK1	PREX1	

down-regulated

CXCL8	PRDX4	PI4K2B	ZKSCAN4	CCNH
AKR1C1	TRG-AS1	TMED3	ATP5A1	LETM2
TNFSF18	CSF2RA	DCUN1D5	RIPK3	SCP2
NTAN1	C1QBP	GINM1	DEPDC7	PRMT3
LYZ	PSMD1	CBWD2	GABPB1	TMEM206
RBM3	C7orf73	PHB2	TAF1B	NEK3
MAN1B1-AS1	RABGAP1L	ACTR10	POP1	PIGC
IGFBP7	NFXL1	ZDHHC6	GLIPR2	TTC19
H3F3AP4	TMA16	SLC25A5	LINC00641	FAM21A
APCDD1	PPP2CA	NRN1	WAC-AS1	SERPINE3

MAPK8	ACOT2
DESI1	CNOT8
TSNAX	ZNF41
COA1	MCPH1
GOLGA5	BRCC3
ATP5B	UBE2K
NUPL2	MTMR10
SLC25A5-AS1	OSBPL1A
SRP54	PRMT5
NAPB	

Supplementary Figure 3: List of genes that are up and downregulated in iDCs cultured on the RPM compared to static conditions, independently of matrix density.

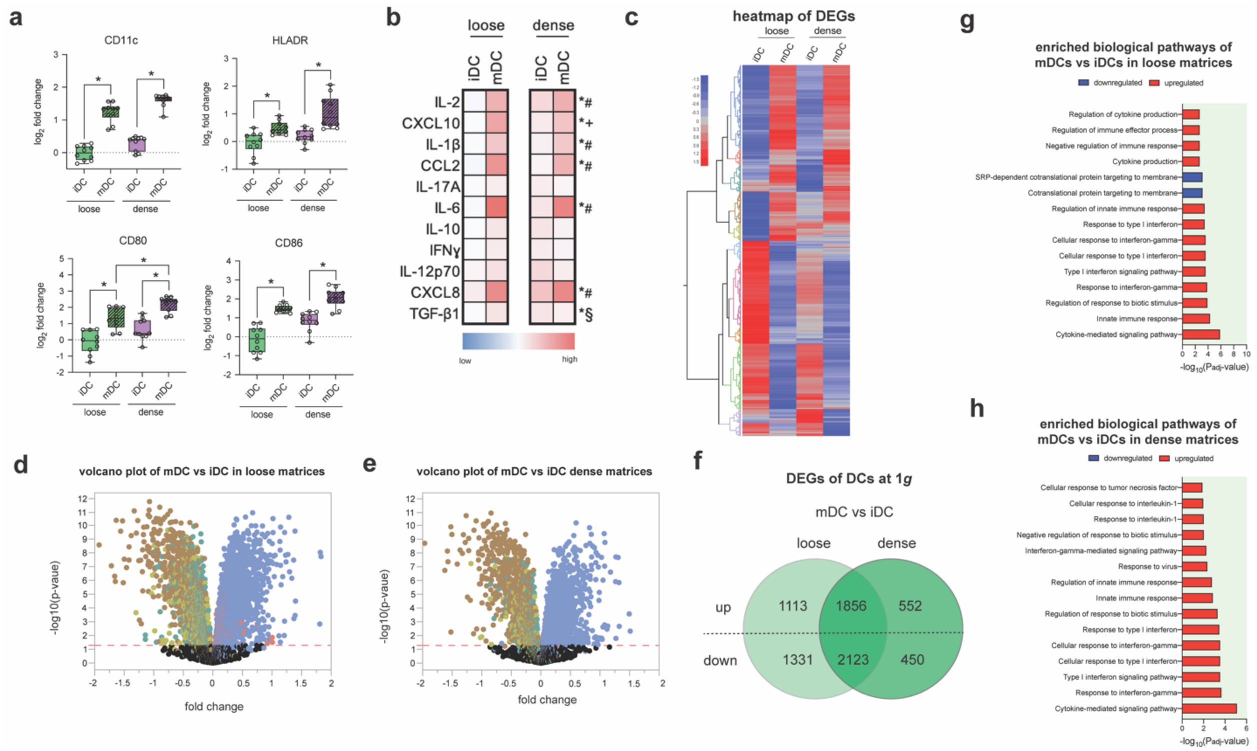

Supplementary Figure 4: Phenotypic and transcriptome analysis of DC differentiation. iDCs and mDCs were cultured for 3 days at static conditions in loose and dense matrices. **a.** Analysis of surface markers associated with differentiation, namely, CD11c, HLADR, CD80, and CD86 using flow cytometry. Log$_2$ fold change of gMFI was calculated relative to iDCs cultured in loose matrices at static conditions. **b.** Heat map of log$_2$ fold change of concentration of cytokine secretion by iDCs and mDCs cultured in loose and dense matrices at static conditions. Log$_2$ fold change of median concentration of cytokine (pg/mL) was calculated relative to cytokine concentration secreted by iDCs in loose matrices at static conditions. **c.** Heatmap of total DEGs in samples after RNA-sequencing analysis. Volcano plots depicting up and downregulated DEGS of mDCs compared to iDCs cultured at static conditions in **d.** loose and **e.** dense matrices compared. **f.** Venn diagram of the number of DEGs of mDCs compared to iDCs cultured in static conditions in either loose or dense matrices. Enriched biological processes pathways in mDCs compared to iDCs cultured in static conditions in **g.** loose and **h.** dense matrices Experiments were performed with at least 3 replicates. The box and whiskers graphs used have the center line at the median value. The upper and lower bounds of the box extend from the 25th to 75th percentiles and the whiskers are plotted to the minimum and maximum values. * indicates significant $p \leq 0.05$. For heatmap, *indicates significant $p \leq 0.05$ of samples in of mDCs in loose matrices compared to iDCs in loose matrices cultured at static conditions. # indicates significant $p \leq 0.05$ of mDCs in dense matrices compared to iDCs in dense matrices cultured at static conditions. § indicates significant $p \leq 0.05$ of iDCs in dense matrices compared to iDCs in loose matrices at static conditions. + indicates significant $p \leq 0.05$ of mDCs in dense matrices compared to mDCs in loose matrices cultured at static conditions.

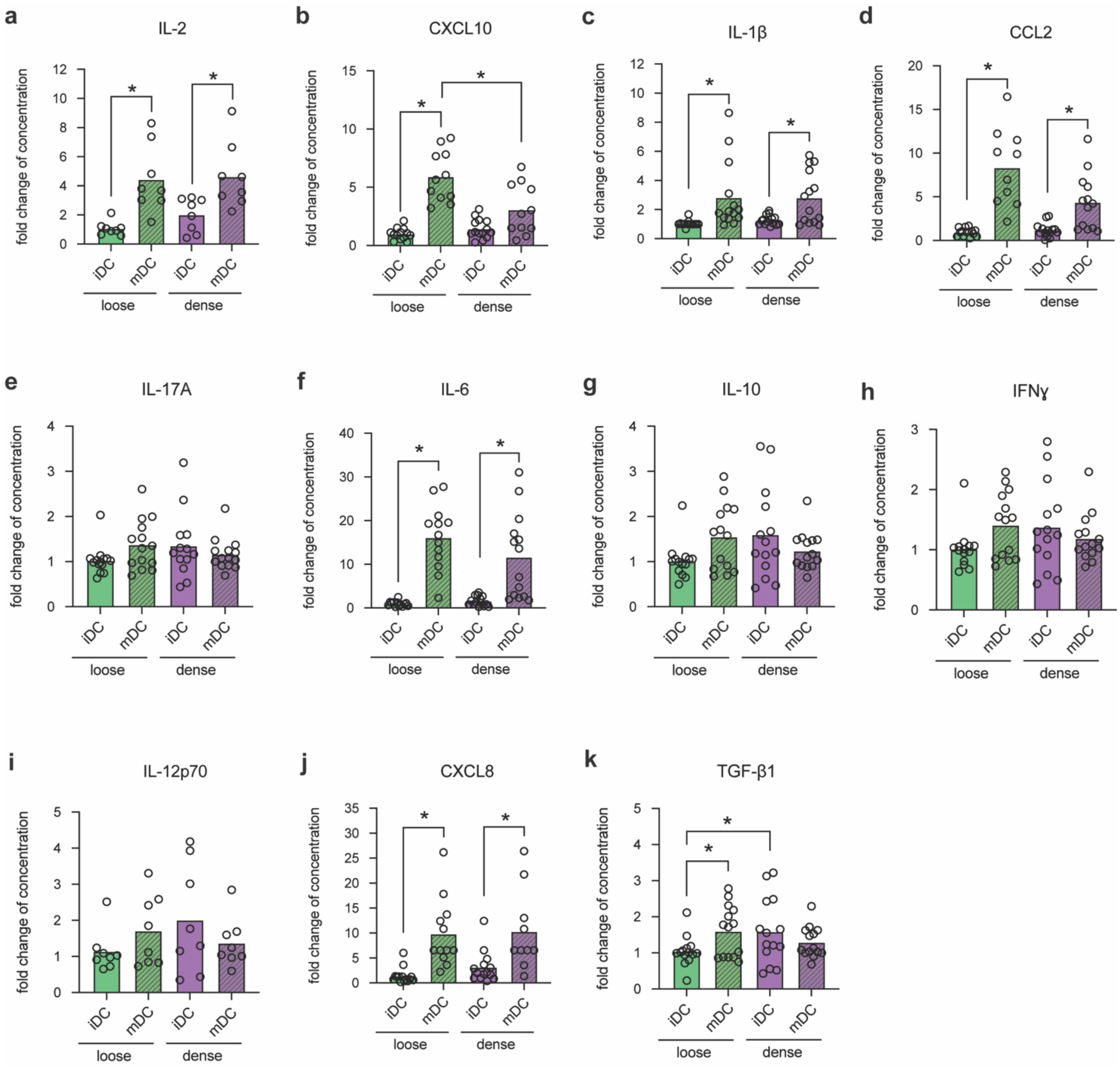

Supplementary Figure 5: Bar graph analysis of cytokine secretion by iDCs and mDCs cultured for 3 days at static or RPM conditions in loose and dense matrices. Fold change of concentration of cytokine (pg/mL) calculated relative to samples cultured in loose matrices at static conditions. Data are shown as mean +/- SD. * indicates significant $p \leq 0.05$.

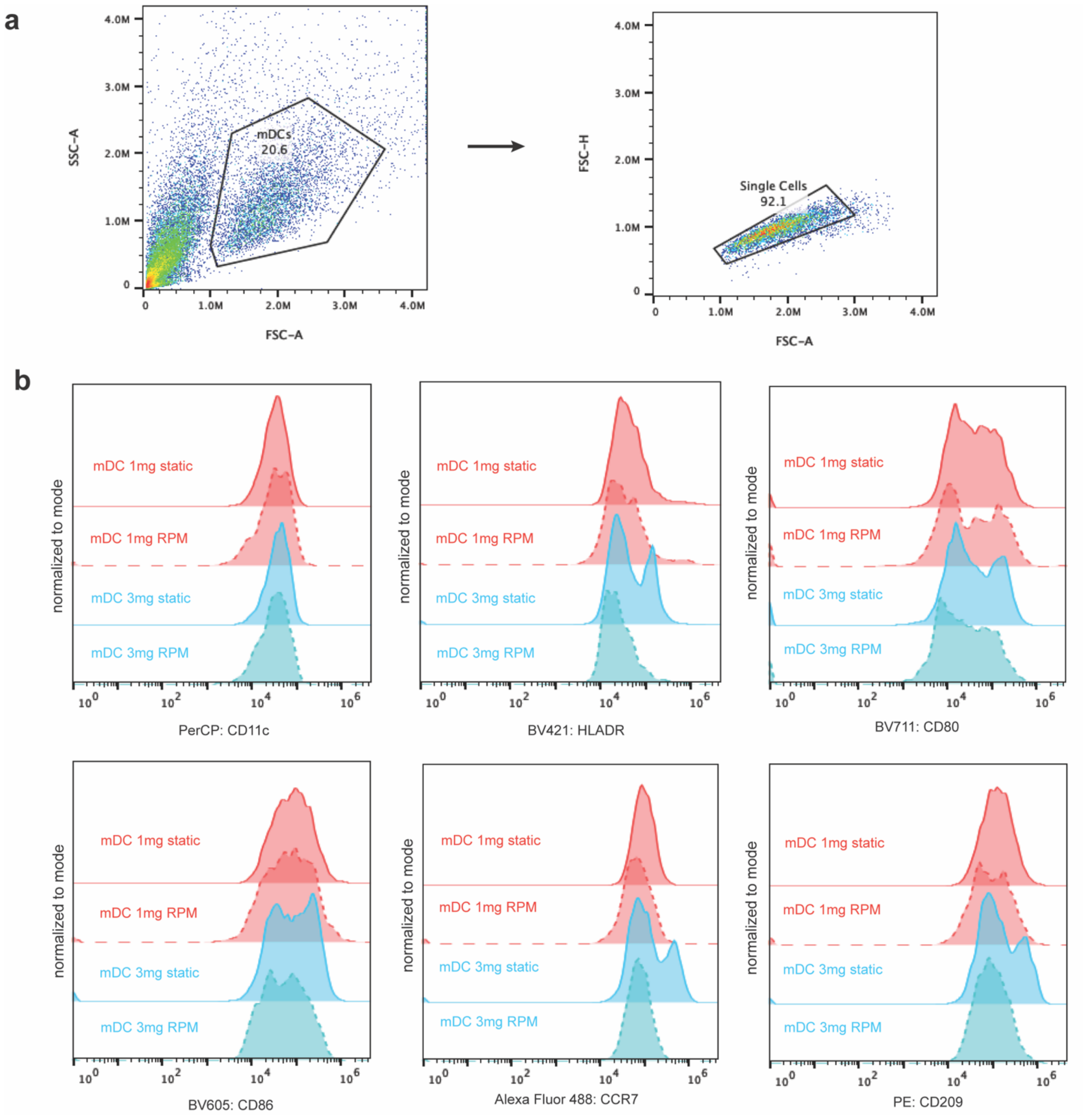

Supplementary Figure 6: a. Representative gating strategy for mDCs cultured under different conditions. Initial cell population was gated on FSC and SSC to remove cell debris and dead cells. The population was then gated to isolate single cells and remove any cell aggregates. This population was then used in the histogram analysis. **b.** Representative fluorescent histograms used in the study. This was used for the data analysis in Figure 5B in the manuscript.

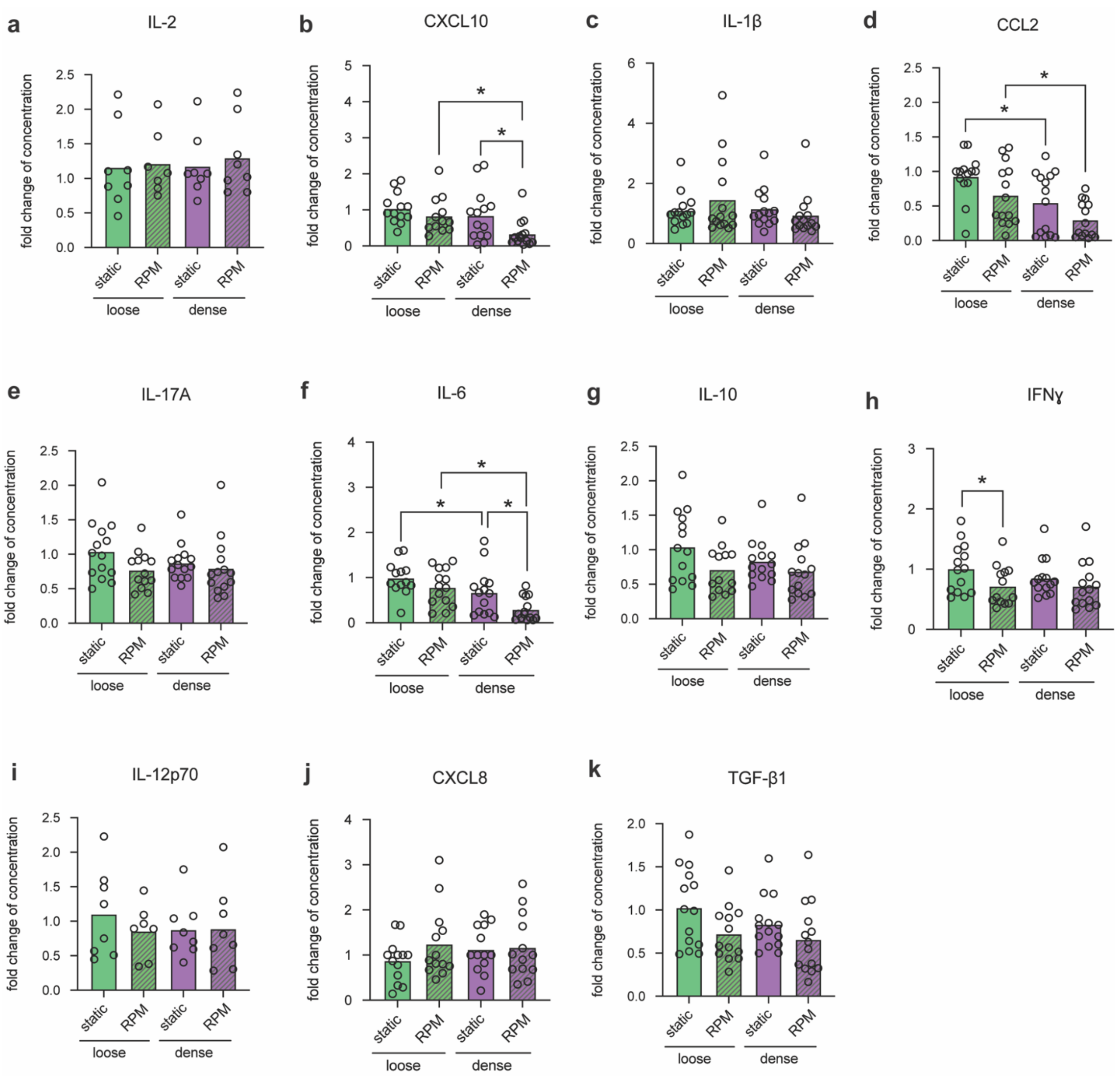

Supplementary Figure 7: Bar graph analysis of cytokine secretion by mDCs cultured for 3 days at static or RPM conditions in loose and dense matrices. Fold change of concentration of cytokine (pg/mL) calculated relative to samples cultured in loose matrices at static conditions. Data are shown as mean +/- SD. * indicates significant $p \leq 0.05$.

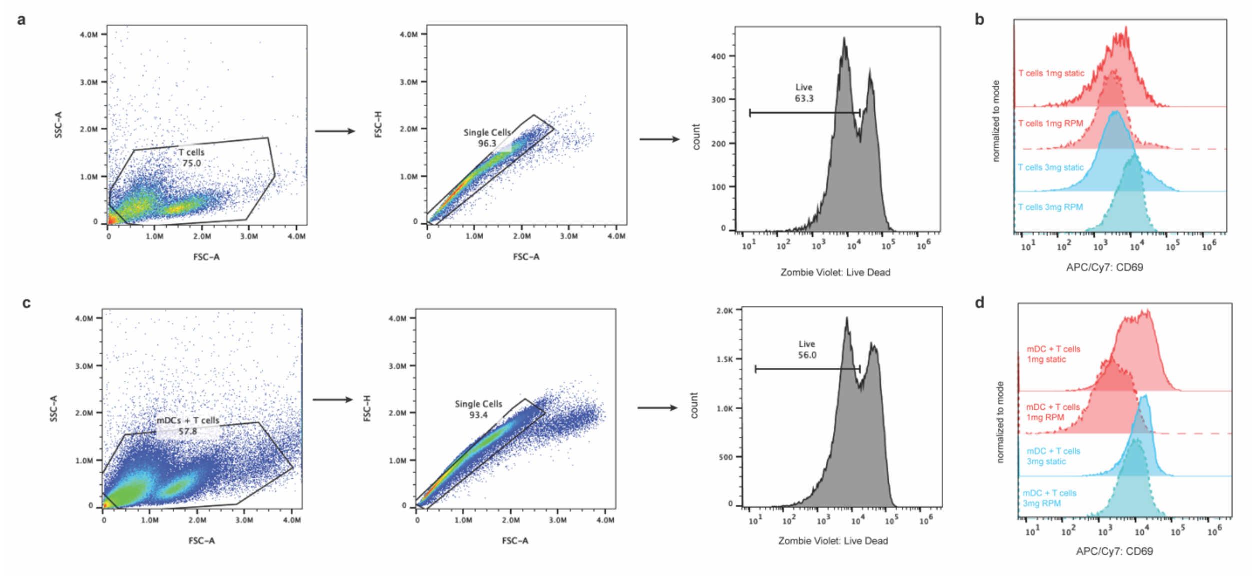

Supplementary Figure 8: a. Representative gating strategy for T cells alone cultured under different conditions. Initial cell population was gated on FSC and SSC to remove cell debris and dead cells. The population was then gated to isolate single cells and remove any cell aggregates. Live cells were then gated using a cell viability dye. This live population was then used in the histogram analysis. **b.** Representative fluorescent histograms of CD69 levels in T cells alone. This was used for the data analysis in Figure 6E in the manuscript. **c.** Representative gating strategy for mDCs and T cells co-cultured under different conditions. Initial cell population was gated on FSC and SSC to remove cell debris and dead cells. Live cells were then gated using a cell viability dye. This live population was then used in the histogram analysis. **d.** Representative fluorescent histograms of CD69 levels in mDCs and T cells that were co-cultured. This was used for the data analysis in Figure 6E in the manuscript.

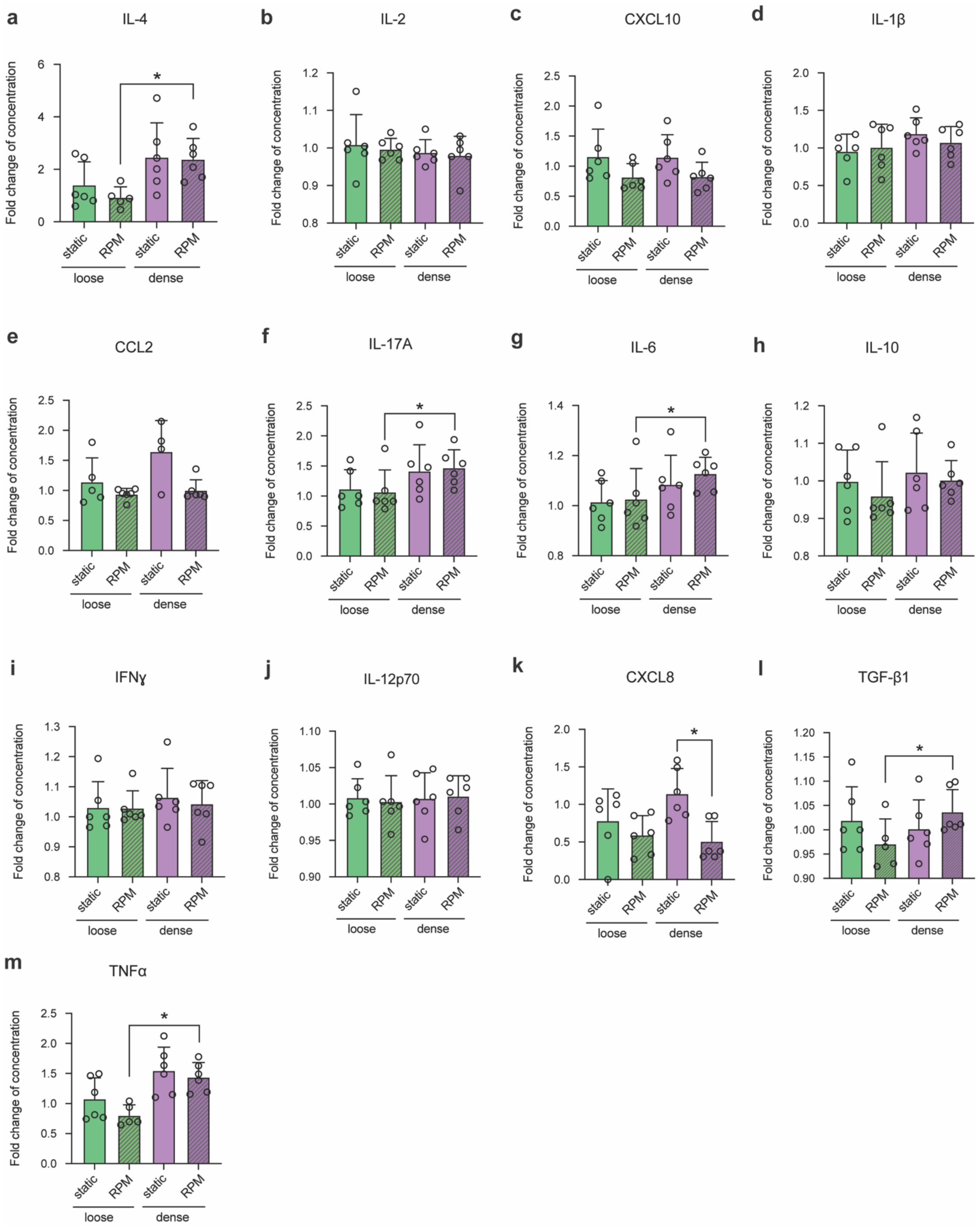

Supplementary Figure 9: Bar graph analysis of cytokine secretion by mDCs co-cultured with T cells for 3 days at static or RPM conditions in loose and dense matrices. Fold change of concentration of cytokine (pg/mL) calculated relative to samples cultured in loose matrices at static conditions. Data are shown as mean +/- SD. * indicates significant $p \leq 0.05$.

up-regulated

OAZ1	CSNK1D	MRPL41	CEBPA-AS1	COL9A2	KLC3	GPC2	DPY19L2P3
MRFAP1	MTHFSD	SLC25A29	ZBTB3	ZNF574	C19orf26	CCDC153	TLX3
BRD9	HDGFRP2	HSPB1	ZNF395	HIP1R	EPHX1	LINC01560	SIX3
CPSF4	STUB1	ARRDC1	C9orf139	COL4A2-AS1	DDIT4	SPRY1	CLDN4
SRSF4	STARD3	TPRA1	ATP8B3	UBALD1	CYB561A3	TRIM73	POU5F1P4
PFN1	RNF167	QTRT1	POLD4	AP4M1	EN2	C10orf91	TMCO2
SARAF	MAP3K14	FLCN	ZSCAN2	BMF	SYTL1	LINC00926	FALEC
CYC1	TNRC6C	PTOV1	UQCC3	SIX5	TCHH	PRR7	OR4K2
RCE1	PWWP2B	FLYWCH1	PEX11A	CEBPB	MESTIT1	ACP5	LINC00853
BCS1L	KDM4A-AS1	BTBD2	FAAH	AACSP1	DACT3	CLK1	POU5F1B
DPP7	MAF1	ZNF74	FAM195A	PTGDS	HOXA7	HSPA2	SLC35G5
PIH1D1	FASTK	CCNE1	SMAD7	SLC16A1-AS1	CTSF	DLK2	POU5F2
RBM10	AP3M2	LRG1	ZNF696	KLF10	PA2G4P4	LINC00304	NKX2-5
PSAP	FBXO32	SLC16A3	LINC01002	CROCC	PPAP2C	C19orf73	IFT74-AS1
RNF149	SMO	SRRM5	U2AF1L4	TMEM150B	GDF7	PSPN	HEPN1
GNPTG	C11orf68	FBXO44	ALDH2	GRN	FBXL22	C9orf172	MTRNR2L4
ATPIF1	RAB11B-AS1	CADM4	PBXIP1	PLA2G7	LRRC8E	S1PR5	FOXN3-AS2
TRIP10	ZNF383	ZNF768	ZNF585A	PEX6	TSSK6	CTGF	VIPR1-AS1
EZR	ZNF740	TYSND1	CHKB	TUBA3FP	DPEP2	CYP1B1	OOEP
TMEM109	RAB11B	SNHG7	TNS3	NPB	AOC1	CAPN10-AS1	RPRML
TSR3	KCNQ1OT1	SLC3A2	ANKZF1	PPAN	FAM43A	DNM1P41	MAS1
ASB1	AIFM3	CDC34	ZNF792	RSRP1	CCDC163P	CENPM	HIST3H2BB
GM2A	CDIPT	TMEM138	ZSWIM1	PPP1R35	DNAJB2	PRSS27	SNORA25
ITPR3	TMEM150A	C19orf25	C1orf35	SCN1B	HIST1H2AC	ACY1	LINC01574
ATP6V1E2	TOMM40	C11orf21	CFP	MKNK2	SH2B1	GADD45G	ZNF847P
SGSM2	C7orf26	SP9	CEBPB-AS1	LINC00899	FAM229A	TNFRSF4	MIR765
CD3EAP	PARP16	TRAF2	PIK3C2B	KCNH2	TNFSF14	SEMA6B	SNORA51
LAGE3	SIRT3	FTH1	KIAA0930	LINC01011	CCR10	KCNJ11	MIR4284
LAPTM5	PTPRS	PPOX	SLC45A4	P3H4	TNFRSF25	RAB26	MIR6085
SMG9	CNPPD1	PTOV1-AS2	DISP2	PRRG3	MAP1A	C16orf82	

down-regulated

FCER1A	UBE2J1	USP53	PLEKHA2	MAN2A1	NT5C2	SLC3A1	ADRBK2	ABCB10
MNDA	TRG-AS1	ACSL4	FAR2	TMEM167B	KIAA0020	LYN	LRRC40	TLDC2
CXCL11	PNPT1	SSR1	GALNT1	REV3L	LYPLA1	ADAM10	PHF20L1	SPOPL
TNFSF18	SLFN11	ACPP	SLC12A6	SLC25A32	SLC35A3	EEA1	PIGX	CPT2
HOPX	NCOA7	CDC42EP3	APOOL	KLHL2	ZNF430	DDX26B	TP53RK	ATL3
HESX1	ITGA4	KDELC2	DNAJC10	EIF5A2	SLC33A1	OSGIN2	CDK17	CBFB
TNFSF10	DUSP6	ST3GAL5	MAGT1	EXOSC9	ADCY6	DOCK11	CDK19	INIP
ENPP2	CYSLTR1	ST6GAL1	CYB5B	SPTLC2	USP14	IFT74	IFNAR2	MESDC2
GPR183	FUT4	B3GNT2	ATP2B1	TMED5	HS2ST1	MTDH	CUL4B	ZNF791
RTP4	NUDT16P1	LRRC8B	LRP12	RPL17	SLC4A7	PCGF5	KCTD9	RAB6A
HPSE	IPCEF1	TES	GOLT1B	POLK	C5orf28	XIAP	GORASP2	ARID4B
GCNT1	LIMA1	PTGES3L	PTP4A1	SLFN12	PANX1	BAZ1A	MAN1A2	TMEM41B
APCDD1	CBWD1	ARL5A	SECISBP2L	ZDHHC21	LCP1	SKAP2	FAM13B	MAPK9
TIFA	TAPT1	TMEM2	NT5C3A	PARP8	PAPD4	GOLGA5	ELOVL5	EPC2
DDX60L	BLOC1S6	LINC00641	CXorf38	EIF2AK3	MTMR10	MOB1B	DEK	ARHGAP26
TXLNB	NRIP3	CCNH	SAV1	ARL4A	ANKRD28	PDLIM5	TWSG1	PSPC1
FCGR1B	GK	GNB4	MBNL1	JAK2	PTPRE	TAB2	ROCK2	PPP3R1
ARRDC3	PROS1	SLC8A1	DNAJC3	METTL21B	HDAC9	YES1	SYDE2	SYNJ1
SASH3	STK17B	MBNL1-AS1	SLC9B2	NDFIP2	ARL8B	GNE	XPNPEP1	FLVCR1
MIR155HG	HNRNPA1P10	PI4K2B	GDAP1	CD55	UCHL5	DHTKD1	USP32	WWC2
CLEC12B	TTC39B	HIF1A	CXorf21	BMP2K	ZNF280D	KLF3	TLK2	TMEM65
TMEM133	AIM1	TDRD7	AZI2	SLC24A1	ZNF561	PDIA4	MUT	AIDA
TRIB1	AMD1	HSD17B12	MGME1	SH3GLB1	CD2AP	C5orf15	AEBP2	HIATL1
RBM3	SGMS2	KIF17	SSR3	TRIM38	MASTL	DENND5A	EML4	KIAA1191
GLUD1P3	LAIR1	SHOC2	CENPL	TMED10	CASS4	LAT2	EZH2	PPP1R12A
KIAA0040	C9orf72	RPL36A	NUDT4	TMEM106B	TGS1	DSC2	XPR1	USP46
SLITRK4	IGFBP7	CHM	PMAIP1	SMCHD1	CD47	RNF139	PPP2R2A	TMTC4
FAM105A	PCM1	GPATCH11	ERLEC1	CRLF3	DSN1	MSL3	LMO2	RAB8A
GBP4	LY75	PPARG	TEX2	MZT1	RB1	XBP1	ZMYM5	
LPCAT2	STARD4	STARD8	ACER3	DNAJC21	METTL4	COA1	NFX1	

Supplementary Figure 10: List of genes that are up and downregulated in mDcs cultured on the RPM compared to static conditions, independently of matrix density.
