# Supplementary material for: Effects of an aged tissue niche on the immune potency of dendritic cells using simulated microgravity
Source: NPJ Aging. 2023 Jul 1;9(1):14. doi: 10.1038/s41514-023-00111-7 (PMC10314949; doi:10.1038/s41514-023-00111-7)
Supplement: Supplementary file 2 — reporting summary [file 41514_2023_111_MOESM2_ESM.pdf]

nature portfolio

Corresponding author(s): Jeremy Teo

Last updated by author(s): May 9, 2023

Reporting Summary

Nature Portfolio wishes to improve the reproducibility of the work that we publish. This form provides structure for consistency and transparency in reporting. For further information on Nature Portfolio policies, see our Editorial Policies and the Editorial Policy Checklist.

Statistics

For all statistical analyses, confirm that the following items are present in the figure legend, table legend, main text, or Methods section.

n/a	Confirmed	
☐	☒	The exact sample size (*n*) for each experimental group/condition, given as a discrete number and unit of measurement
☐	☒	A statement on whether measurements were taken from distinct samples or whether the same sample was measured repeatedly
☐	☒	The statistical test(s) used AND whether they are one- or two-sided<br>*Only common tests should be described solely by name; describe more complex techniques in the Methods section.*
☒	☐	A description of all covariates tested
☐	☒	A description of any assumptions or corrections, such as tests of normality and adjustment for multiple comparisons
☒	☐	A full description of the statistical parameters including central tendency (e.g. means) or other basic estimates (e.g. regression coefficient) AND variation (e.g. standard deviation) or associated estimates of uncertainty (e.g. confidence intervals)
☒	☐	For null hypothesis testing, the test statistic (e.g. *F*, *t*, *r*) with confidence intervals, effect sizes, degrees of freedom and *P* value noted<br>*Give P values as exact values whenever suitable.*
☒	☐	For Bayesian analysis, information on the choice of priors and Markov chain Monte Carlo settings
☒	☐	For hierarchical and complex designs, identification of the appropriate level for tests and full reporting of outcomes
☒	☐	Estimates of effect sizes (e.g. Cohen's *d*, Pearson's *r*), indicating how they were calculated

Our web collection on statistics for biologists contains articles on many of the points above.

Software and code

Policy information about availability of computer code

Data collection	No custom code was used for collection of data.
Data analysis	Data analyses from multiplex cytokine kit was performed using provided LEGENDplex™ data analysis software (Biolegend, USA), RNA sequencing analysis was done using NASQAR toolbox (publicly accessible at http://nasqar.abudhabi.nyu.edu/; accessed on 20 March 2022), JMP Genomics (JMP®, Version <9>. SAS Institute Inc., Cary, NC, 1989–2021) software, and Idep96 (http://bioinformatics.sdstate.edu/idep96/; accessed on 17th June 2022). Analysis of T cell infiltration into collagen gels was done using a custom-built MATLAB script (MATLAB R2019b; MathWorks Inc., USA) that can be provided upon request.

For manuscripts utilizing custom algorithms or software that are central to the research but not yet described in published literature, software must be made available to editors and reviewers. We strongly encourage code deposition in a community repository (e.g. GitHub). See the Nature Portfolio guidelines for submitting code & software for further information.

Data

Policy information about availability of data

All manuscripts must include a data availability statement. This statement should provide the following information, where applicable:

- Accession codes, unique identifiers, or web links for publicly available datasets
- A description of any restrictions on data availability
- For clinical datasets or third party data, please ensure that the statement adheres to our policy

The datasets generated and/or analyzed from RNA seq during the current study are available and can be accessed

Field-specific reporting

Please select the one below that is the best fit for your research. If you are not sure, read the appropriate sections before making your selection.

☒ Life sciences ☐ Behavioural & social sciences ☐ Ecological, evolutionary & environmental sciences

For a reference copy of the document with all sections, see nature.com/documents/nr-reporting-summary-flat.pdf

Life sciences study design

All studies must disclose on these points even when the disclosure is negative.

Sample size	For all experiments n=3 was chosen as the minimal replicate number. We determined this to be sufficient owing to appropriate experimental controls for each particular set of experiments.<br>Surface marker analyses, n=10<br>Cytokine quantification, n>=5<br>OVA uptake analysis via flow cytometry, n>=9<br>Gene expression analyses (RNAsequencing), n=3<br>Cell migration analyses, n=3 and at least 4 different positions were volumetrically imaged for quantification.<br>Co-culture experiments, n=6
Data exclusions	Data were not excluded from analyses.
Replication	No replication studies were performed.
Randomization	As the study did not involve any human or animal subjects and the treatment samples were being compared under tightly controlled conditions, randomization was deemed unnecessary.
Blinding	Blinding was not implemented during the experiments, and the data collected was measurable such that blinding would not have an impact on any bias in the collected data.

Reporting for specific materials, systems and methods

We require information from authors about some types of materials, experimental systems and methods used in many studies. Here, indicate whether each material, system or method listed is relevant to your study. If you are not sure if a list item applies to your research, read the appropriate section before selecting a response.

Materials & experimental systems

n/a	Involved in the study	
☐	☒	Antibodies
☐	☒	Eukaryotic cell lines
☒	☐	Palaeontology and archaeology
☒	☐	Animals and other organisms
☒	☐	Human research participants
☒	☐	Clinical data
☒	☐	Dual use research of concern

Methods

n/a	Involved in the study	
☒	☐	ChIP-seq
☐	☒	Flow cytometry
☒	☐	MRI-based neuroimaging

Antibodies

Antibodies used	1. mouse anti-human CD11c monoclonal antibody; clone: Bu15; conjugated with PerCP; Biolegend 337234; lot: B305074<br>2.mouse anti-human CCR7 monoclonal antibody; clone: G043H7; conjugated with Alexa Fluor 488; Biolegend 353206; lot: B277021<br>3. mouse anti-human HLADR monoclonal antibody; clone L243; conjugated with Brilliant Violet 421; Biolegend 307636; lot: B311370<br>4. mouse anti-human CD80 monoclonal antibody; clone 2D10; conjugated with Brilliant Violet 711; Biolegend 305236; lot: B315414<br>5. mouse anti-human CD86 monoclonal antibody; clone BU63; conjugated with Brilliant Violet 605; Biolegend 374214; lot: B354057<br>6. mouse anti-human CD206 monoclonal antibody; clone 15-2; conjugated with Brilliant Violet 510; Biolegend 321138; lot: B322915<br>7. mouse anti-human CD209 monoclonal antibody; clone 9E9A8; conjugated with PE; Biolegend 330106; lot: B266379<br>8. mouse anti-human CD69 monoclonal antibody; clone FN50; conjugated with APC/Cyanine7; Biolegend 310914; lot:B360870
Validation	As manufacturer's website, antibody clones are tested in a variety of assays to see which applications they are suited for: https://www.biolegend.com/en-us/quality/product-development<br>1. https://www.biolegend.com/en-us/products/percp-anti-human-cd11c-antibody-12734?GroupID=BLG6213<br>2. https://www.biolegend.com/en-us/products/alexa-fluor-488-anti-human-cd197-ccr7-antibody-7496?GroupID=BLG9611<br>3. https://www.biolegend.com/en-us/products/brilliant-violet-421-anti-human-hla-dr-antibody-7226<br>4. https://www.biolegend.com/nl-nl/products/brilliant-violet-711-anti-human-cd80-antibody-15864<br>5. https://www.biolegend.com/en-us/products/brilliant-violet-605-anti-human-cd86-antibody-15822?GroupID=GROUP28

6. https://www.biolegend.com/en-us/products/brilliant-violet-510-anti-human-cd206-mmr-antibody-15366
7. https://www.biolegend.com/en-us/products/pe-anti-human-cd209-dc-sign-antibody-4885?GroupID=BLG5849
8. https://www.biolegend.com/en-us/products/apc-cyanine7-anti-human-cd69-antibody-1917?GroupID=BLG10036

Eukaryotic cell lines

Policy information about cell lines

Cell line source(s)	1. THP1: AddexBio; cell line C0003024, Lot:1589326<br>2. Jurkat: AddexBio; cell line C0003039; clone E6-1
Authentication	Cell were authenticated prior to purchase
Mycoplasma contamination	1. Negative from source; Cells were tested for mycoplasma using MycoAlert mycoplasma detection kit.<br>2. Negative from source; Cells were not tested for mycoplasma contamination
Commonly misidentified lines<br>(See ICLAC register)	None of the cell types used are listed in the ICLAC database.

Flow Cytometry

Plots

Confirm that:

☒ The axis labels state the marker and fluorochrome used (e.g. CD4-FITC).

☒ The axis scales are clearly visible. Include numbers along axes only for bottom left plot of group (a 'group' is an analysis of identical markers).

☐ All plots are contour plots with outliers or pseudocolor plots.

☒ A numerical value for number of cells or percentage (with statistics) is provided.

Methodology

Sample preparation	Cells were removed from within collagen matrices by digesting with collagenase (6mg/mL) for 10 mins at 37oC. Cells were then stained with appropriate antibodies for 30 mins on ice at 1:250 concentration.
Instrument	Cytek Aurora flow cytometer (4L 16V-14B-10YG-8R)
Software	FlowJo software ((Becton, Dickinson and Company, NJ, USA)
Cell population abundance	An initial gate was used to ensure a cell count of at least 20,000 cells (events) per sample.
Gating strategy	Cell populations were first gated for a live population using FSC and SSC plot to remove cell debris and dead cells (small FSC v SSC) and large clumps or aggregates of cells (large FSC or SSC) and used across all samples. This live population was then used in fluorescent histograms for the different cell surface markers.

☒ Tick this box to confirm that a figure exemplifying the gating strategy is provided in the Supplementary Information.
